# Supplementary material for: Prolonged hydrogen production by engineered green algae photovoltaic power stations
Source: Nat Commun. 2023 Oct 25;14:6768. doi: 10.1038/s41467-023-42529-3 (PMC10600337; doi:10.1038/s41467-023-42529-3)
Supplement: Supplementary file 1 — Supplementary Information [file 41467_2023_42529_MOESM1_ESM.pdf]

Prolonged hydrogen production by engineered green algae photovoltaic power stations

Gwon *et al.*

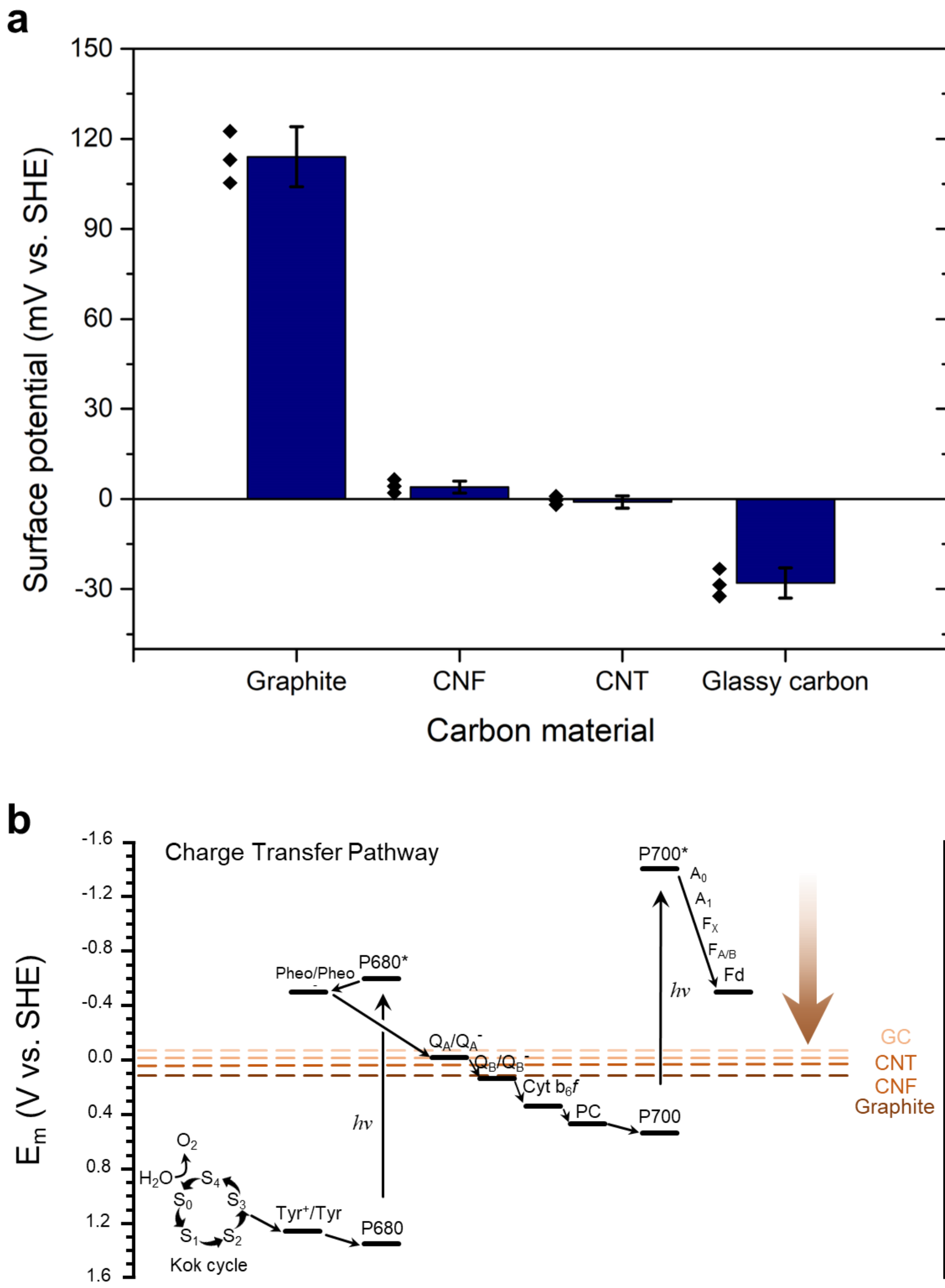

Supplementary Figure 1. Surface potentials of various carbon materials for photoelectron harvesting. (a) Measured (zeta-potential analyzer) surface potentials of graphite, carbon nanofiber (CNF), carbon nanotube (CNT), and glassy carbon (GC) were 114 mV, 4 mv, -1 mV, -28 mV (vs. SHE), respectively. (b) Midpoint potentials of electron carriers in the thylakoidal Z-scheme. All carbon materials tested exhibit appropriate surface potentials for photoelectron extraction. CNF was chosen owing to the proper morphology for the insertion surgery into algae. Data are presented as mean values ± SD, error bars indicate standard deviations (n = 3, independent samples). Source data are provided as a Source Data file.

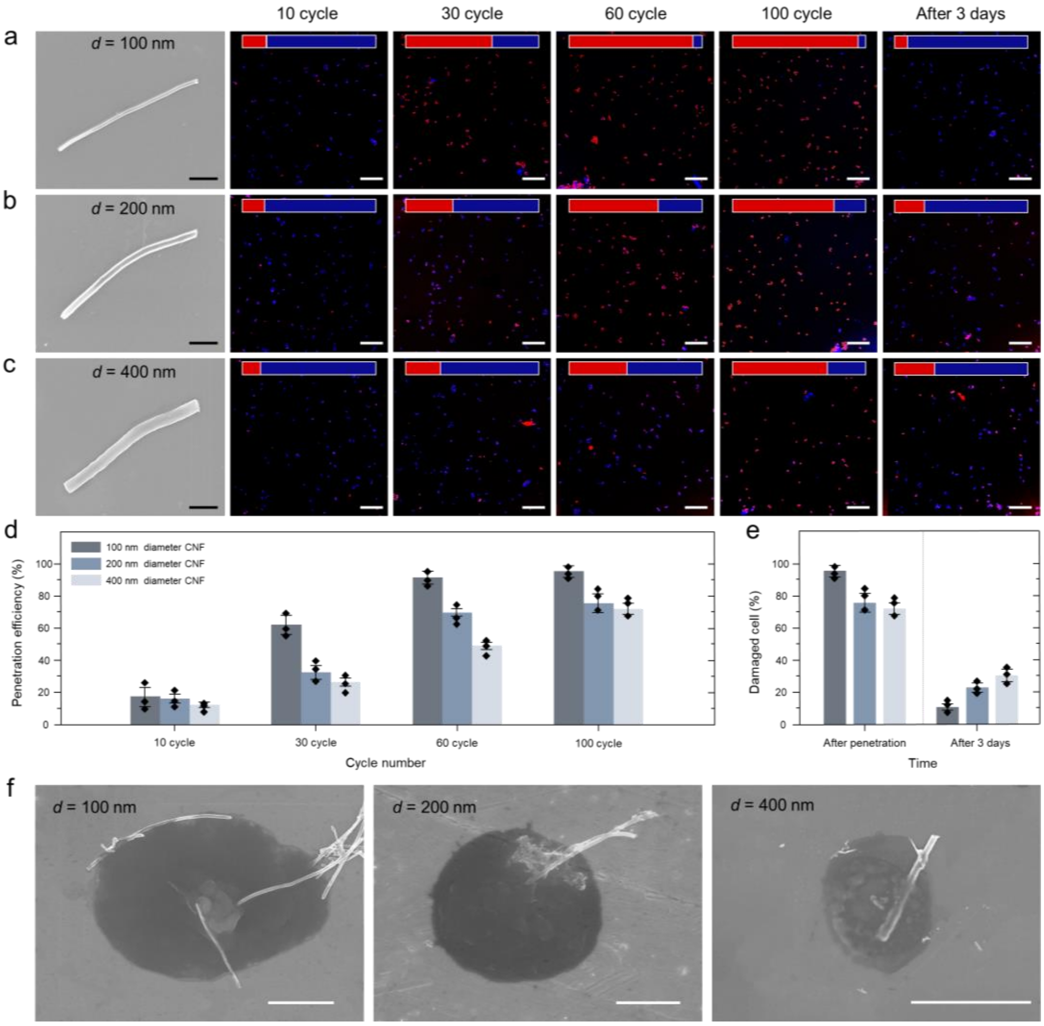

Supplementary Figure 2. Experimental validation of *C. reinhardtii* membrane penetration by CNFs. (a to c) Left: SEM images of carbon nanofibers with end diameters of (a) 100 nm, (b) 200 nm and (c) 400 nm. Right: CLSM overlay projections of nonpenetrated (blue fluorescent) and penetrated (red fluorescent) *C. reinhardtii* on three different CNFs. Corresponding percentages of nonpenetrated and penetrated cells are plotted in the images in the bar graph on the top of the image. The CLSM image at the right end shows regeneration of the cell walls 3 days after the insertion surgery. Scale bars: 1 µm in SEM images and 100 µm in CLSM images. (d) Bar graphs showing penetration efficiencies at varying number of insertion cycles for the three types of CNFs. Optimal conditions yielded 94% penetration efficiency. (e) Fraction of cells exhibiting penetration (damaged, left) after the surgery and those after 3 days of recovery (right). Most cells recover fully in 3 days when inserted with 100 nm end diameter CNFs. (f) Representative SEM images of *C. reinhardtii* inserted with 100 nm diameter CNFs, 200 nm diameter CNFs, and 400 nm diameter CNFs. Data in (d-e) are presented as mean values ± SD, error bars indicate standard deviations (n = 3, biologically independent samples). All relevant experiments were performed independently in triplicate with similar results. Source data are provided as a Source Data file.

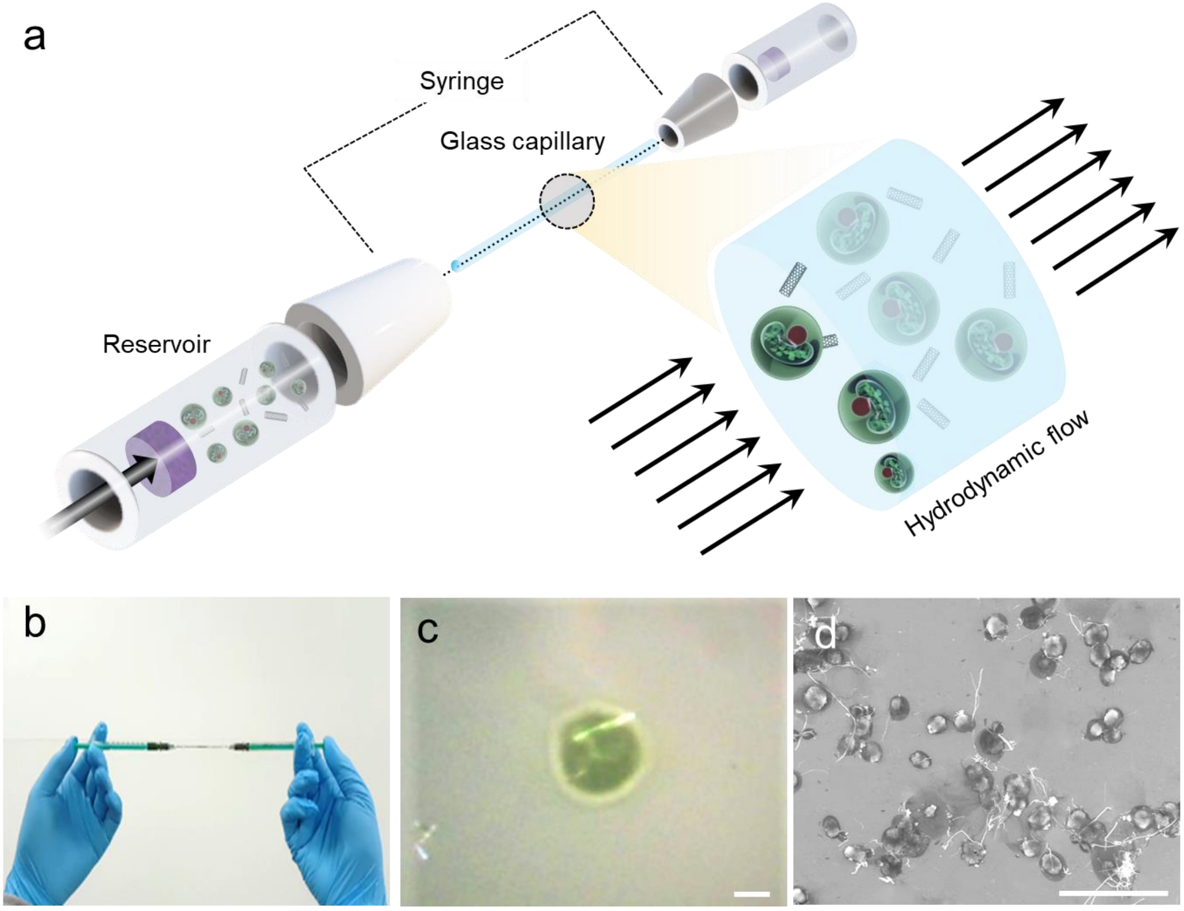

Supplementary Figure 3. Scheme of CNF insertion surgery. Electron and optical micrographs of CNFs inserted *C. reinhardtii*. (a) Schematic diagram of CNFs insertion surgery into algae. A hydrodynamic flow was formed inside a glass capillary to induce effective collisions between the cells and CNFs. (b) Picture of an actual experiment. The number of round trips was between 50 and 100 cycles. (c) Optical image of *C. reinhardtii* inserted with a 100 nm end diameter CNF. (d) Low magnification SEM image of CNFs inserted *C. reinhardtii*. Scale bars in (c) is 5 μm and (d) is 50 μm. All relevant experiments were performed independently in triplicate with similar results.

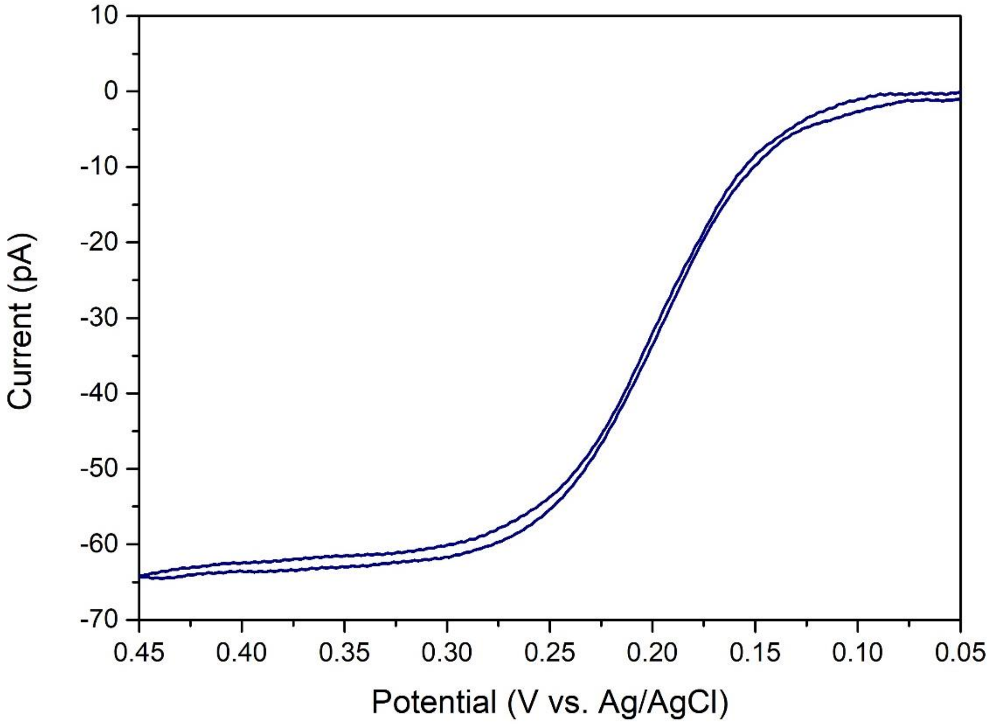

Supplementary Figure 4. Cyclic voltammogram of a platinum nanoelectrode SECM tip. The area of the electrochemically activated electrode was estimated with the oxidation of 1 mM ferrocenemethanol in a 0.1 M phosphate buffer solution. The steady-state current was -64 pA, indicating that the electrode diameter was 425 nm (scan rate 50 mV/s).[51] Source data are provided as a Source Data file.

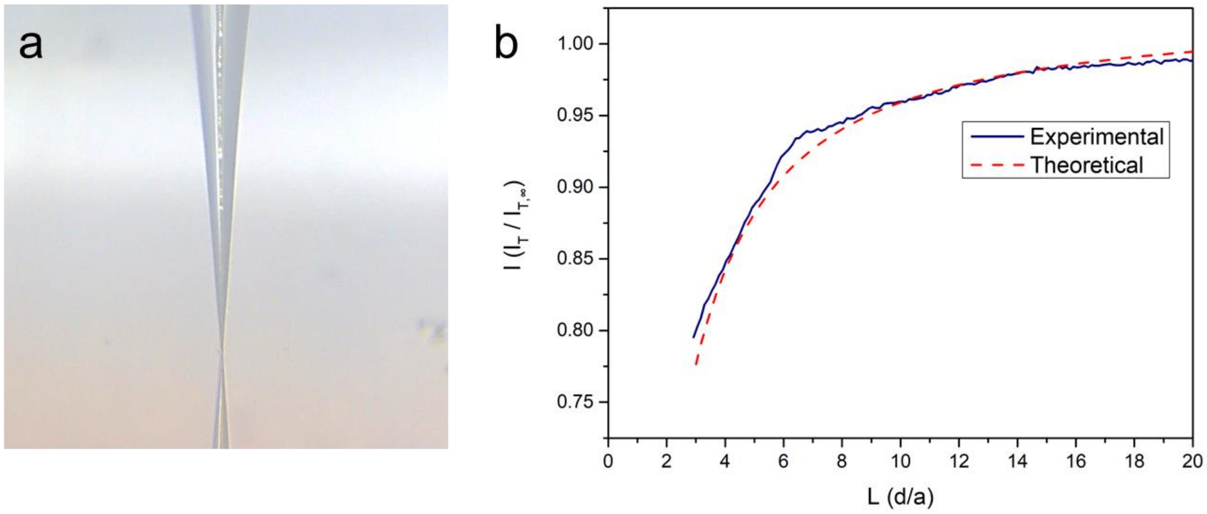

Supplementary Figure 5. SECM tip approach for photosynthetic current detection on a single cell power station. (a) Video microscope (acA2500-14uc, BASLER, Germany) image of cell access of platinum nanoelectrode. (b) Experimental current-distance curve and corresponding theoretical fit obtained with platinum nanoelectrode approaching a single cell photovoltaic power station.[36] The distance between the SECM tip and the engineered cell was *ca.* 1 µm. Experiment was held at 1 mM ferrocenemethanol in 0.1 M phosphate buffer solution. Source data are provided as a Source Data file.

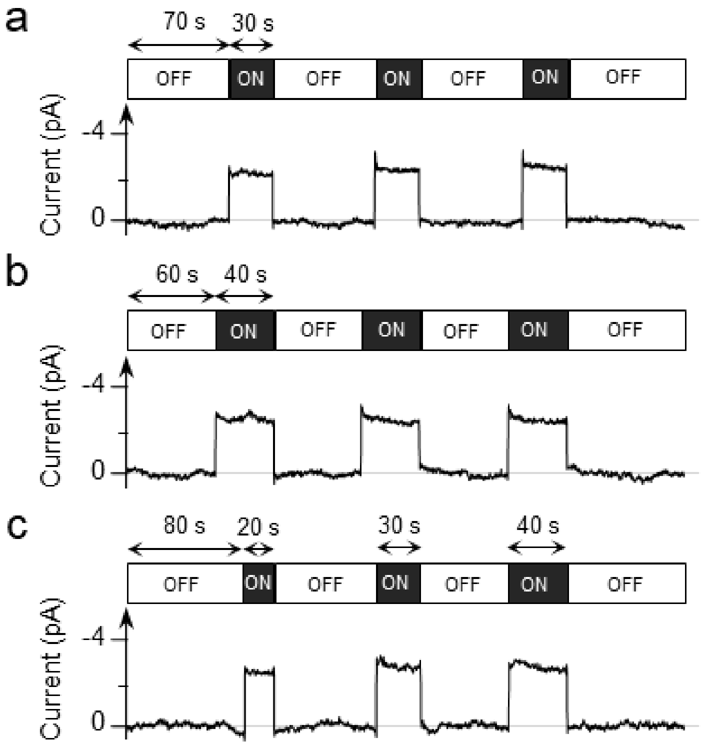

Supplementary Figure 6. Intermittent-light chronoamperogram of the *C. reinhardtii* – CNF. Current response corresponding to the presence of light was observed. Light intensity was 39 µmol photons·m^{-2}·s^{-1}. Chronoamperograms shown in series with (a) 30 s light pulses, (b) 40 s light pulses, and (c) mixed length pulses ranging between 20 and 40 s. Source data are provided as a Source Data file.

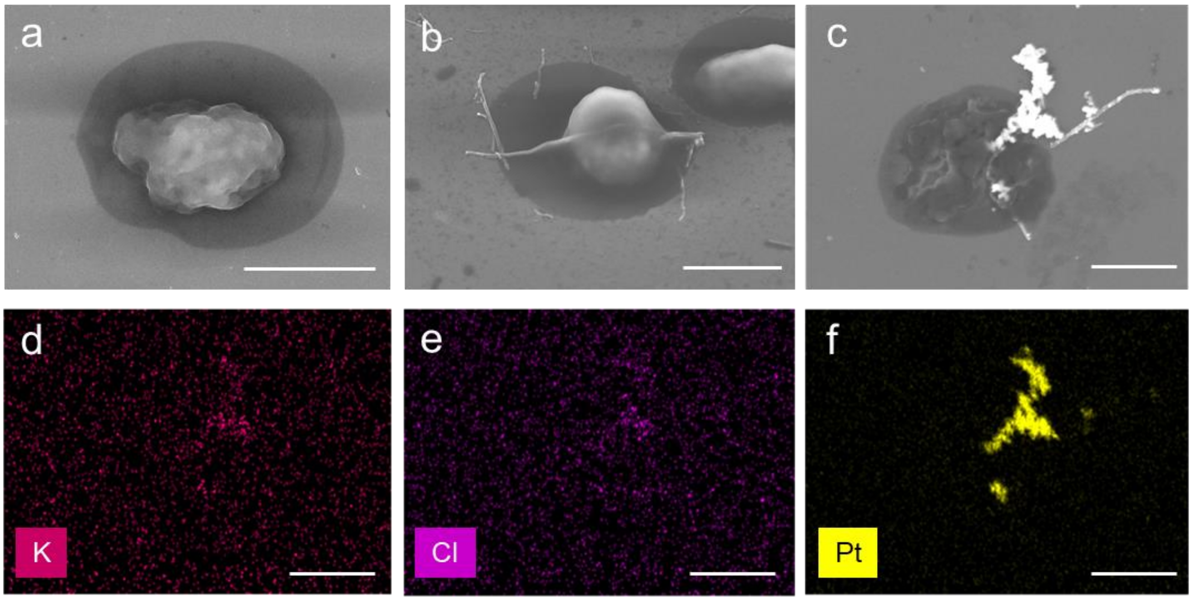

Supplementary Figure 7. Additional electron microscope images of *C. reinhardtii* - CNF/Pt. (a) SEM image of CNFs free *C. reinhardtii.* (b) SEM image of a CNF inserted cell. (c) SEM image of *C. reinhardtii* - CNF/Pt. EDS maps of (d) potassium (e) chlorine (f) platinum elements. Specific Pt electrodeposition occurred on the CNF surface, as opposed to a non-specific adsorption of the precursor, as indicated by the absence of the K and Cl element from the precursor molecule K_2PtCl_4. All scale bars are 5 μm in length. All relevant experiments were performed independently in triplicate with similar results.

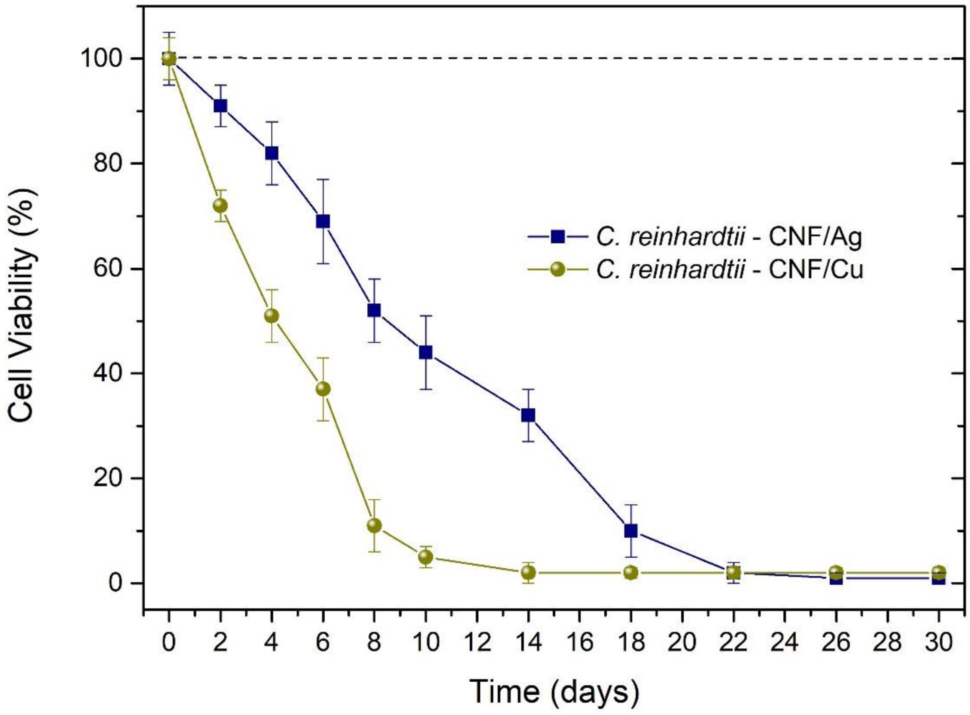

Supplementary Figure 8. Cell viability of *C. reinhardtii* - CNF/Ag and *C. reinhardtii* - CNF/Cu cultures. In *C. reinhardtii* - CNF/Ag culture, cell viability fell below 50 % after 8 days. In *C. reinhardtii* - CNF/Cu cultures, the cell viability fell below 50 % after 4 days. Even after that, rapid cell death rates were seen, indicating that the cytotoxicities of the metal particles were high. Data are presented as mean values ± SD, error bars indicate standard deviations (n = 3, biologically independent samples). Source data are provided as a Source Data file.

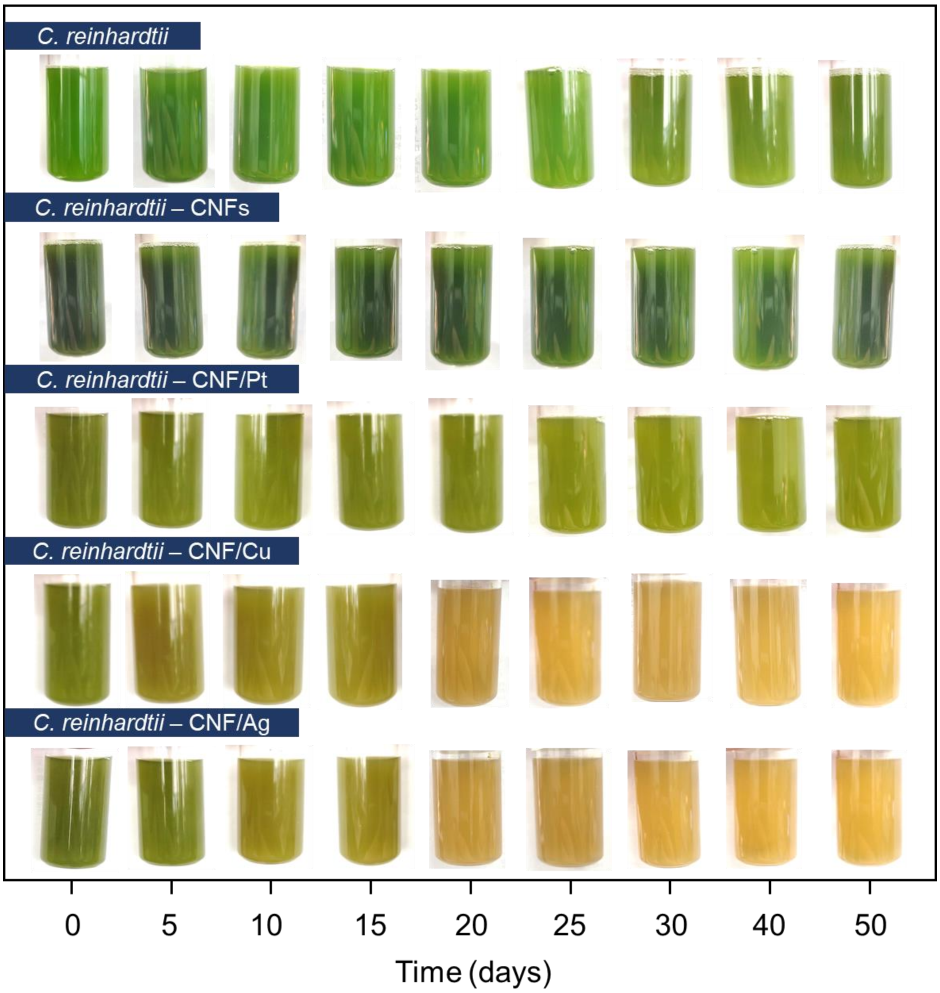

Supplementary Figure 9. Photographs of *C. reinhardtii* culture systems. From the top row, *C. reinhardtii* culture tube, *C. reinhardtii* - CNF culture tube, *C. reinhardtii* - CNF/Pt culture tube, *C. reinhardtii* - CNF/Cu culture tube, *C. reinhardtii* - CNF/Ag culture tube. Cellular photosynthetic activity can be confirmed through color change of cell culture. Photographs taken on different days up to 50 days. Viability confirmed for *C. reinhardtii*, *C. reinhardtii* - CNF, and *C. reinhardtii* - CNF/Pt. All relevant experiments were performed independently in triplicate with similar results.

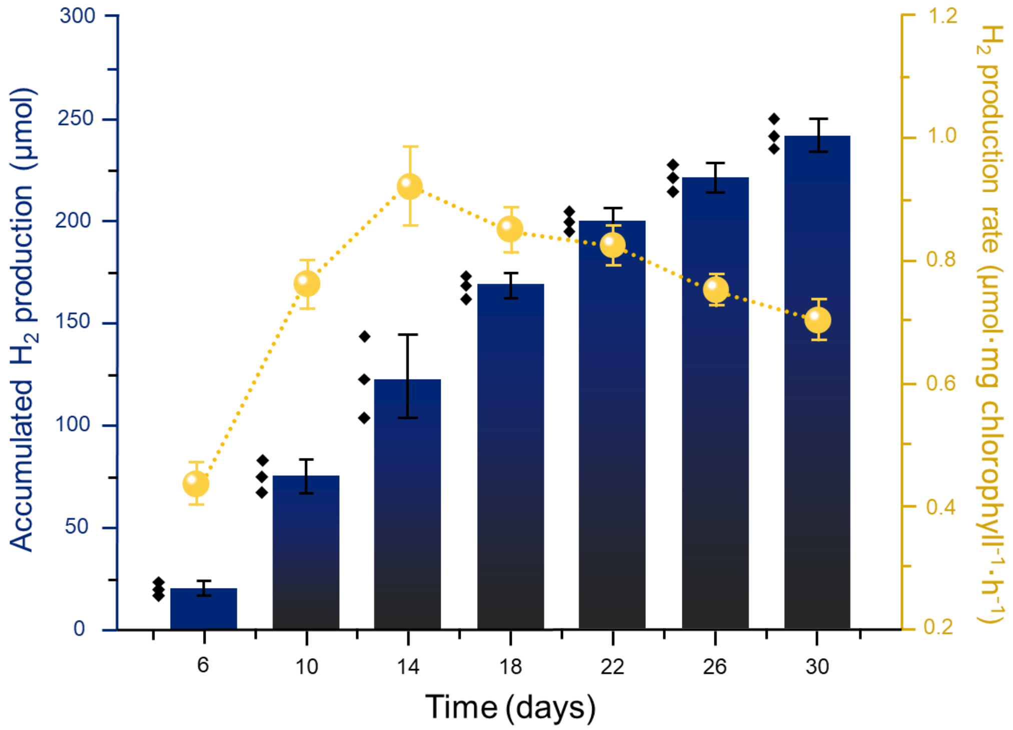

Supplementary Figure 10. H_2 production in TAP media without solution replenishment. Experiments were performed under no TAP growth media change and with cultures adjusted to same chlorophyll content. Decline in the rate of H_2 production was observed after 14 days due to the full consumption of solution acetate and headspace CO_2, resulting in effective starvation of the culture. Data are presented as mean values ± SD, error bars indicate standard deviations (n = 3, biologically independent samples). Source data are provided as a Source Data file.

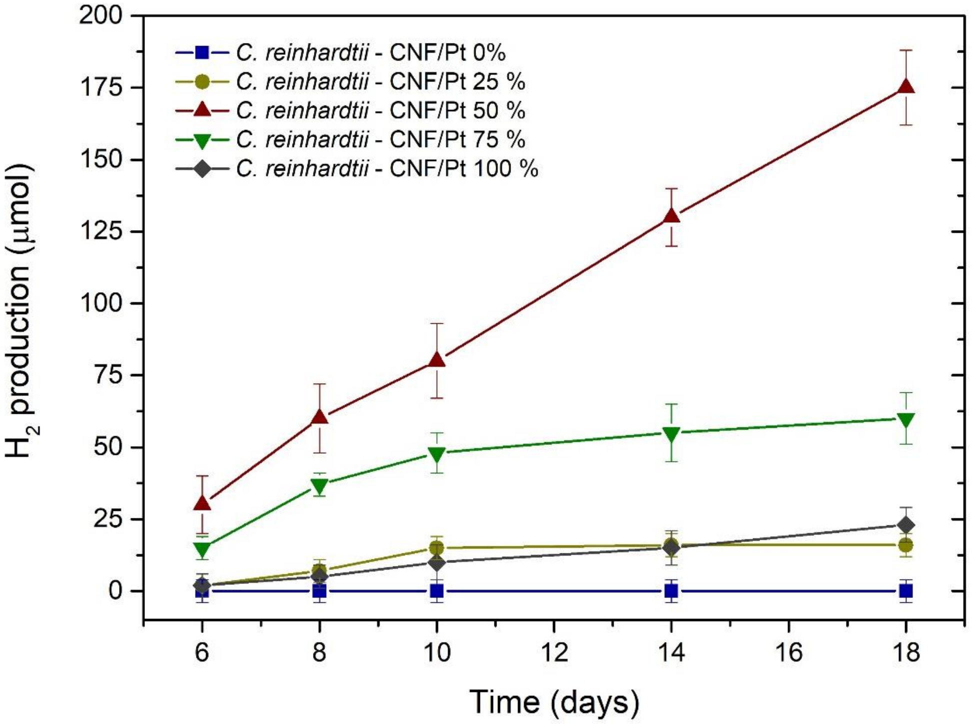

Supplementary Figure 11. H_2 production with various ratios of engineered and pristine algae. Hydrogen production efficiency was the highest when engineered and pristine algae were in one-to-one ratio. Data are presented as mean values ± SD, error bars indicate standard deviations (n = 3, biologically independent samples). Source data are provided as a Source Data file.

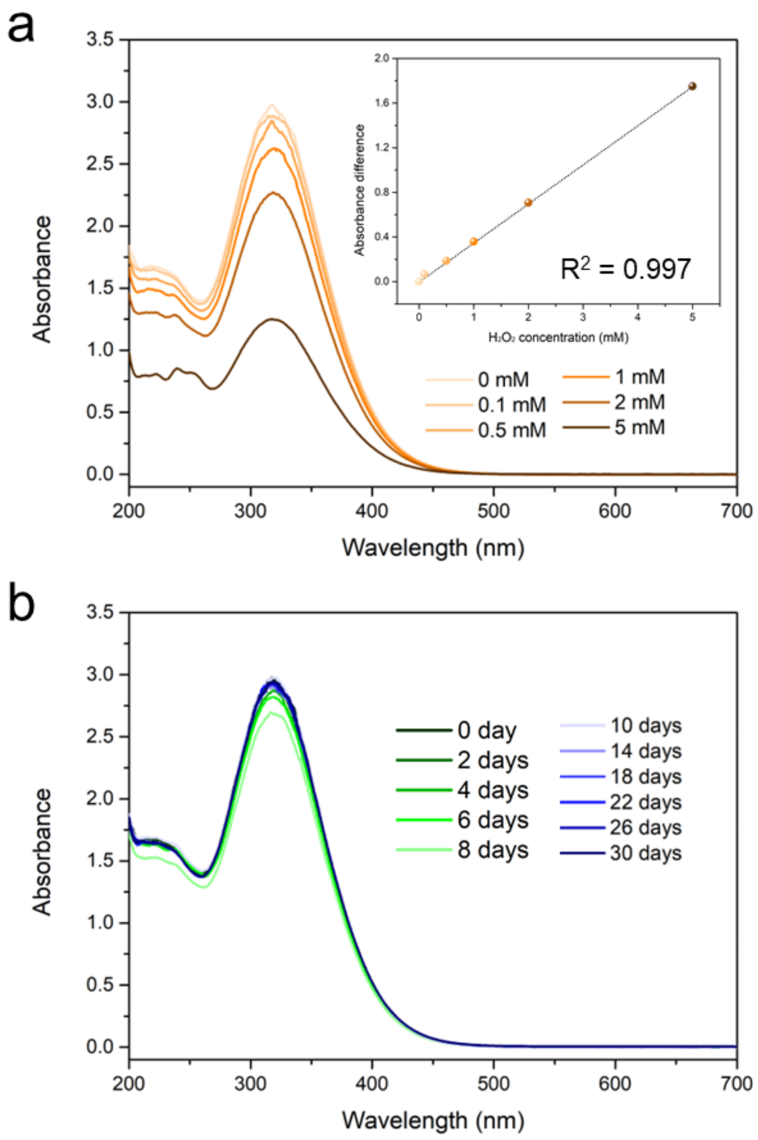

Supplementary Figure 12. Determination of H_2O_2 concentration using cerium sulfate method. (a) A series of control experiments to generation calibration curves for the detection of H_2O_2 by the cerium sulfate method. (b) The change in Ce^{4+} concentration over time could be estimated through the absorption spectrum. The color of the solution changes from yellow to colorless through the reaction. The concentration of Ce^{4+} after the reaction was measured by ultraviolet-visible spectroscopy (JASCO V-770, UV-vis spectrophotometer, Japan) at 316 nm. The number of moles of H_2O_2 in the culture was determined from the peak absorbance and the calibration curve.

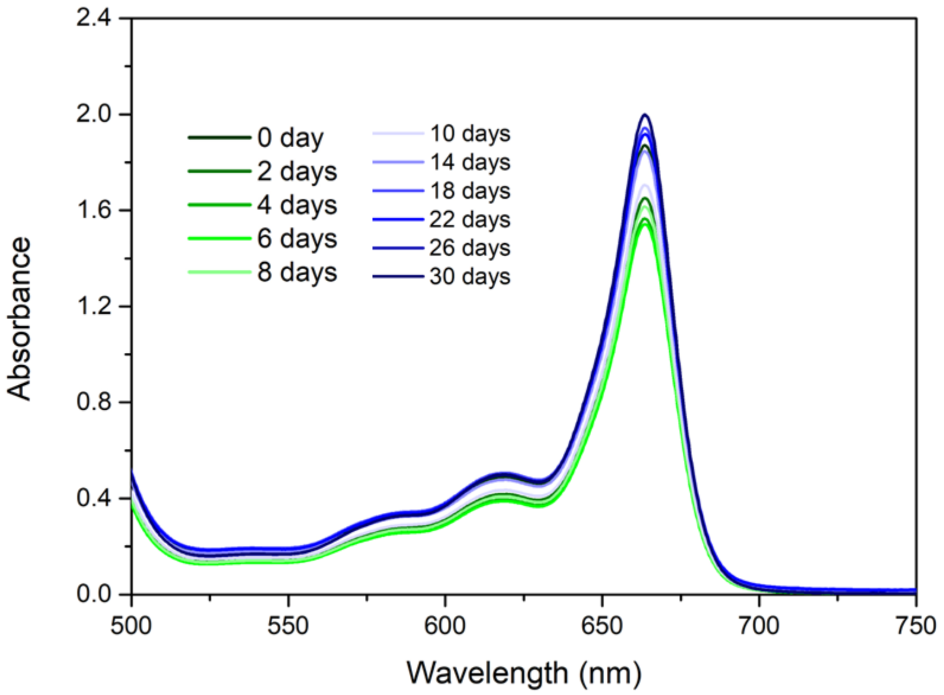

Supplementary Figure 13. Determination of chlorophyll content by UV-vis absorption spectroscopy. The change in chlorophyll concentration over time could be estimated through the absorption spectrum. Absorbance values at 665 nm and 649 nm were used to obtain calculated total chlorophyll concentration (see Methods for calculations).

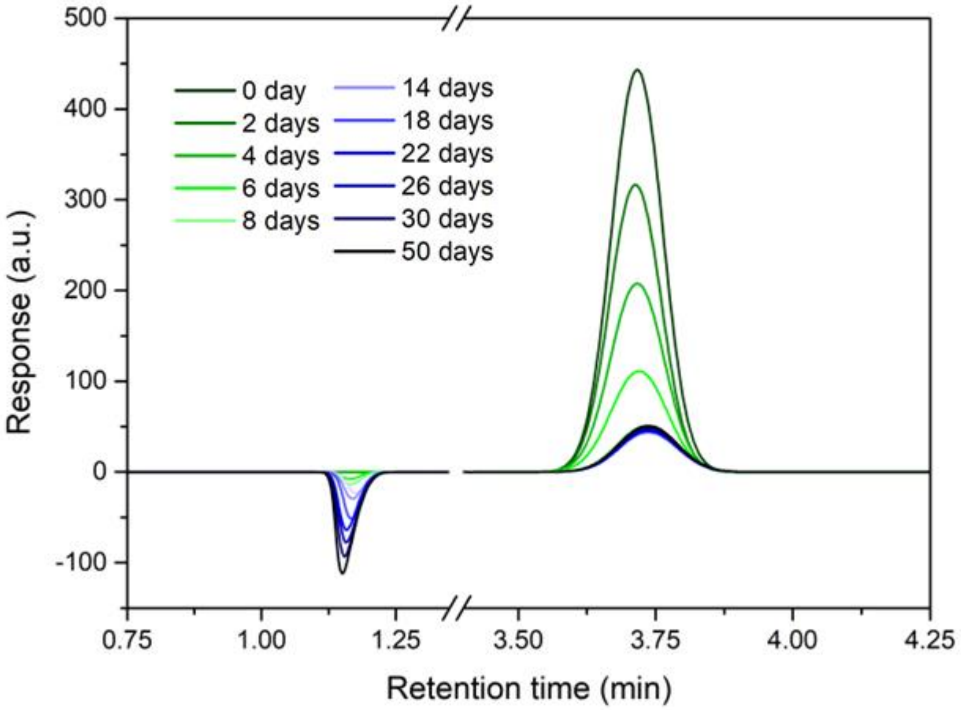

Supplementary Figure 14. Gas chromatogram of *C. reinhardtii* - CNF/Pt culture headspaces. The peak at retention time 1.15 corresponds to H_2, and the peak at 3.7 is for O_2. O_2 decreased until day 8, and H_2 concentration increased from day 6 onwards. The number of moles of gas in the headspace was determined from the peak area.

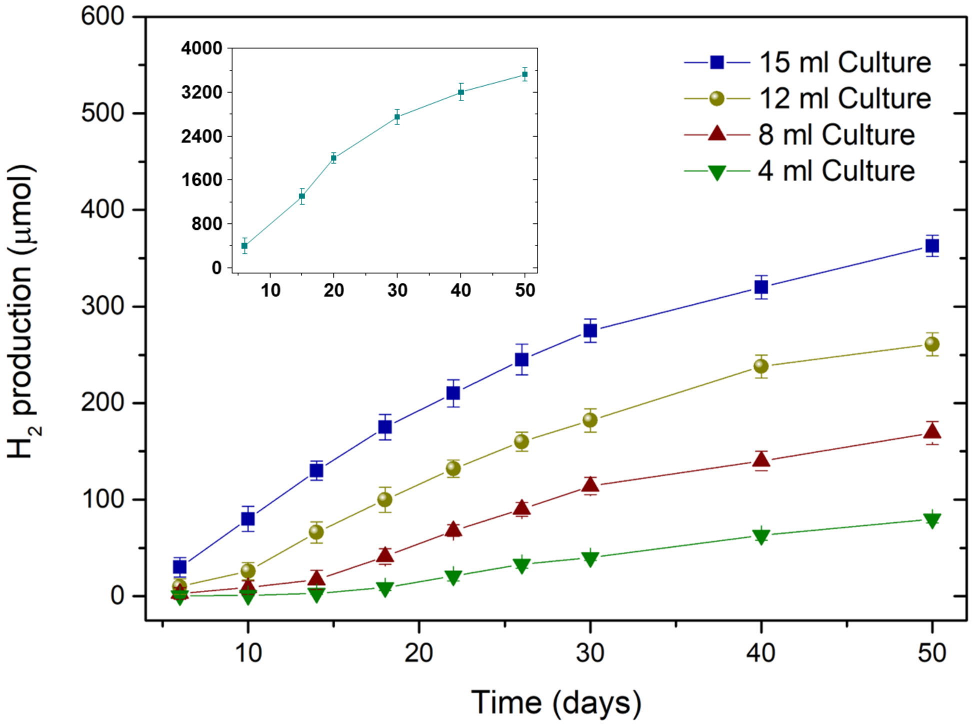

Supplementary Figure 15. H_2 production as a function of cell culture batch reactor volume. The volume of the cell culture was set to 15, 12, 8, and 4 ml, and hydrogen production in the headspace was observed for 50 days. The inset graph shows hydrogen production for 150 ml volume of cell culture. It was confirmed that hydrogen production increased linearly as a function of the batch reactor volume. Data are presented as mean values ± SD, error bars indicate standard deviations (n = 3, biologically independent samples). Source data are provided as a Source Data file.

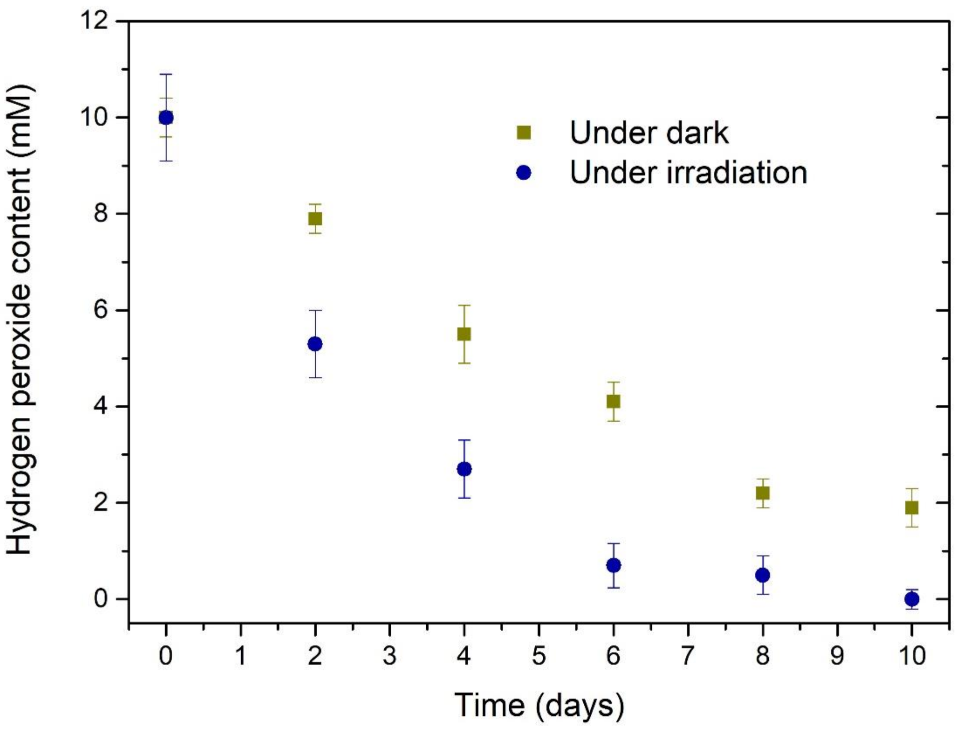

Supplementary Figure 16. Measurement of the decomposition rate of hydrogen peroxide in TAP growth medium. Initially, the concentration of hydrogen peroxide was 10 mM, and then spontaneously decomposed for 10 days at T = 298.15 K. The presence of irradiation affects the rate of degradation. The H_2O_2 decomposition rate obtained from the data shown above was implemented for the mass balance analyses in Figure 5. Data are presented as mean values ± SD, error bars indicate standard deviations (n = 3, biologically independent samples). Source data are provided as a Source Data file.

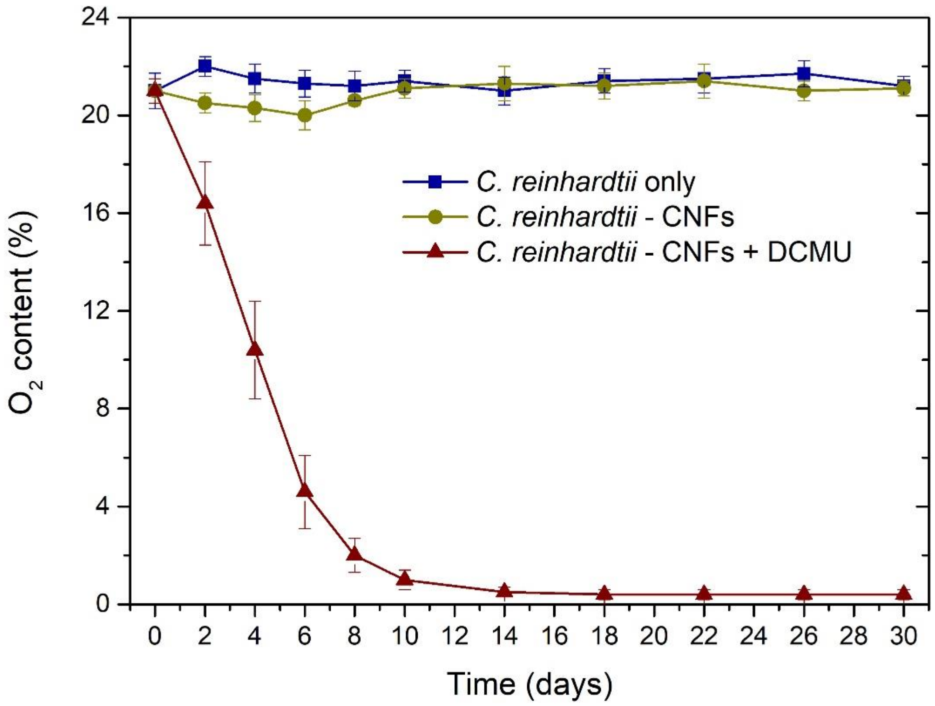

Supplementary Figure 17. Measurement of the O_2 content in the headspace of various cultures. In both *C. reinhardtii* and the *C. reinhardtii* - CNF cultures, the concentration of oxygen was kept constant for 30 days. To measure oxygen consumption due to cellular respiration under illumination, excess of DCMU that interferes with photosystem II was added. In batches with DCMU added, photosynthetic oxygen production was suppressed, and residual oxygen was consumed by cellular respiration. Oxygen in the headspace was continuously consumed for about 10 days, and the oxygen concentration remained low in the presence of DCMU. Oxygen consumption rates by respiration employed in the mass balance analyses (Figure 5) were obtained from the data shown above. Data are presented as mean values ± SD, error bars indicate standard deviations (n = 3, biologically independent samples). Source data are provided as a Source Data file.

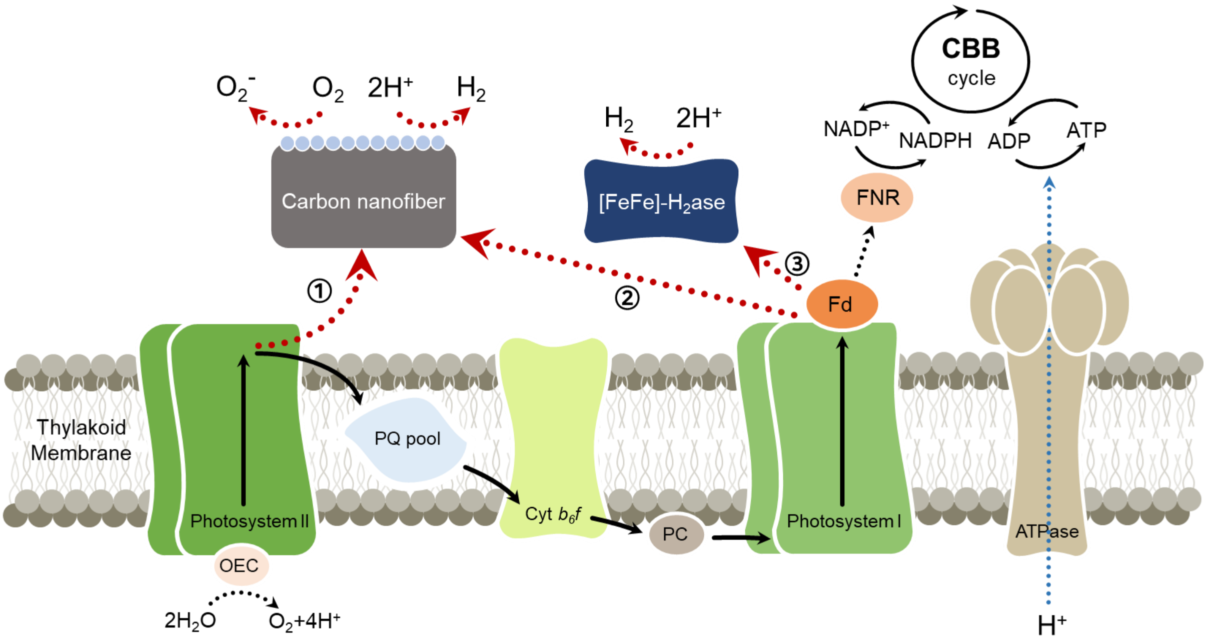

Supplementary Figure 18. Proposed mechanism of electron transfer pathways for *C. reinhardtii* - CNF/Pt. The CNF was inserted into *C. reinhardtii* chloroplasts proximal to thylakoid membranes, where they collected high-energy electrons from either the plastoquinone (PQ, acceptor side of PSII) pool or reduced ferredoxin (Fd, acceptor side of PSI), and transferred them through CNFs. The numbers 1-3 show the pathway of photosynthetic electrons for O_2 consumption and H_2 production. Depending on the residual time and potential of photosynthetic electrons in each photosynthetic component, the probability of electron extraction varies for each scenario.

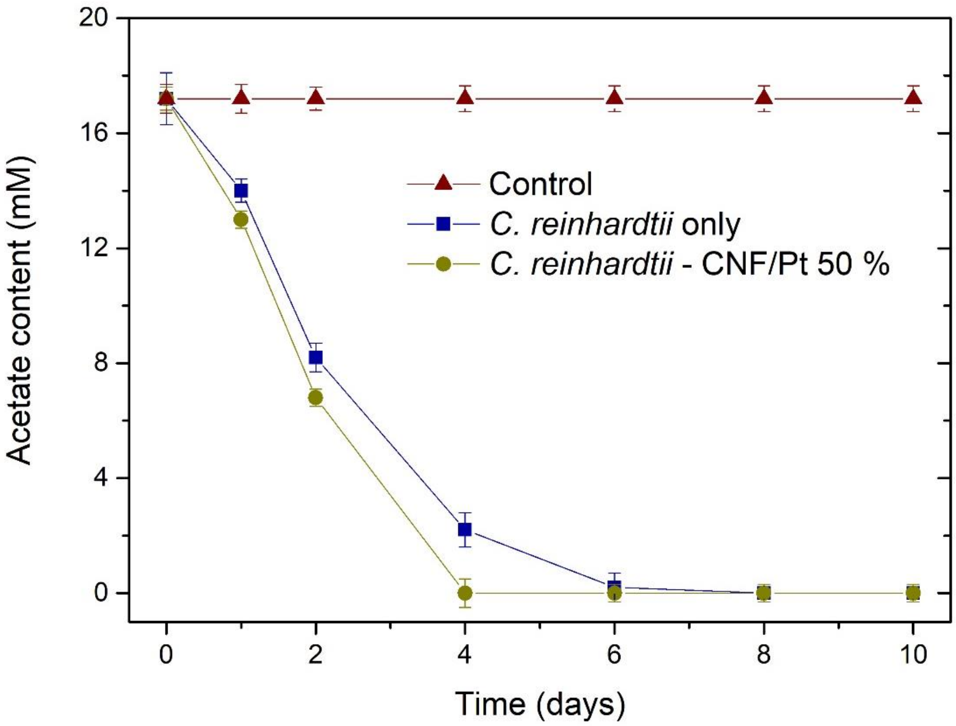

Supplementary Figure 19. Changes over time in the acetate concentration of *C. reinhardtii* - CNF/Pt cultures. The initial concentration of acetate was 17.2 mM, and thereafter it steadily decreased, and both experimental groups consumed all of the acetate after 6 days. Acetate concentration did not decrease during the same period in the control experiment. Control composed of the TAP medium without algae. Data are presented as mean values ± SD, error bars indicate standard deviations (n = 3, biologically independent samples). Source data are provided as a Source Data file.

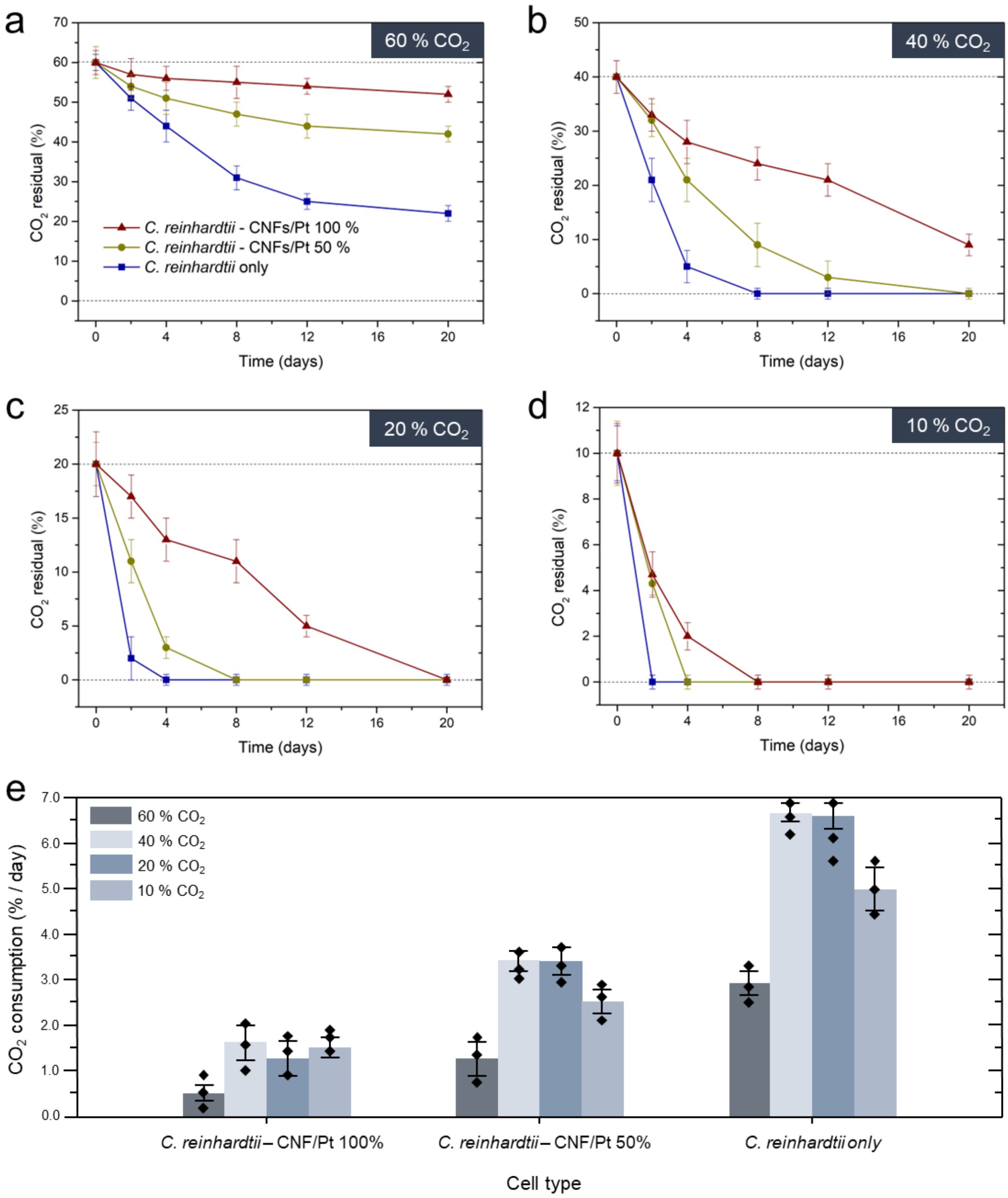

Supplementary Figure 20. Headspace CO_2 consumption in *C. reinhardtii* - CNF/Pt cultures. Dynamic evolution of the headspace CO_2 concentrations in (a) a batch with headspace charged with 60% CO_2, (b) 40% CO_2, (c) 20% CO_2, and (d) 10% CO_2. (e) Summary of CO_2 consumption per day as a function of the ratio between engineered and pristine algae. Data are presented as mean values ± SD, error bars indicate standard deviations (n = 3, biologically independent samples). Source data are provided as a Source Data file.

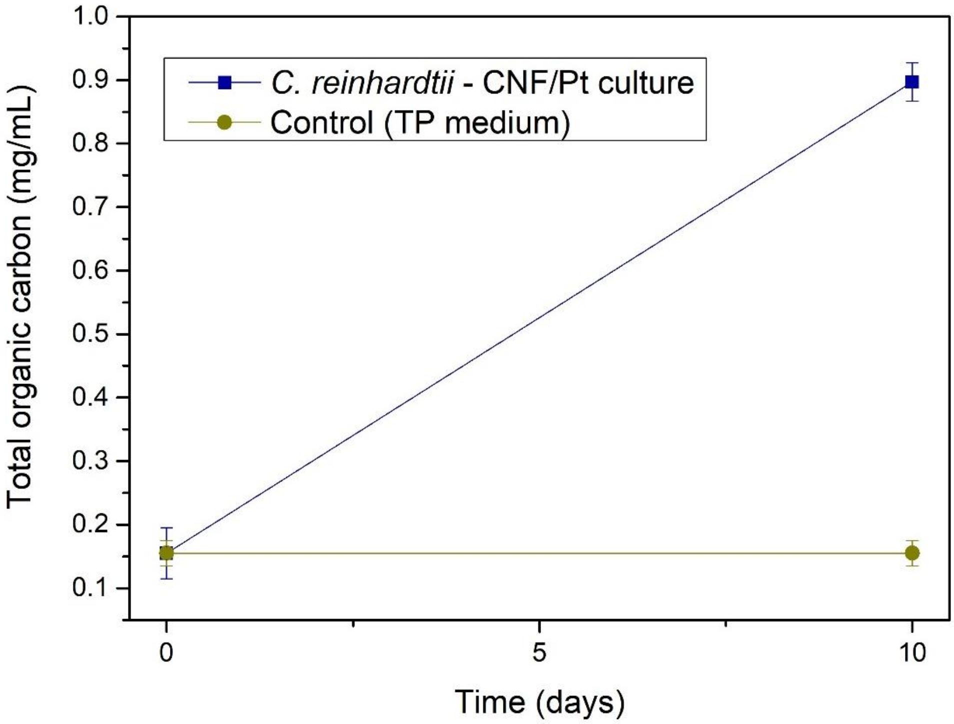

Supplementary Figure 21. Total oxidizable carbon tracking in TP (acetate-free) growth medium. Experiments in acetate-free TP medium to confirm cell lysate composed of organic carbon. TOC increases from 0.16 mg/mL initially to 0.9 mg/mL after 10 days. Data are presented as mean values ± SD, error bars indicate standard deviations (n = 3, biologically independent samples). Source data are provided as a Source Data file.

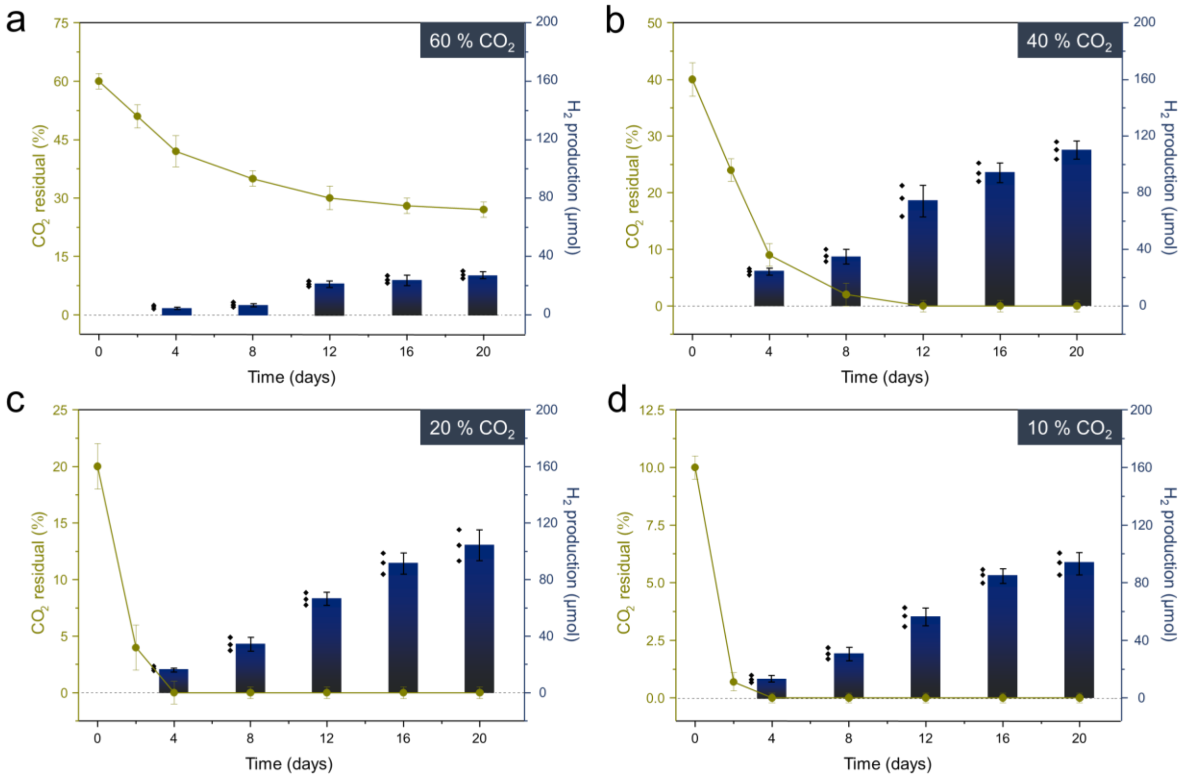

Supplementary Figure 22. Headspace CO_2 consumption and H_2 production in TP (acetate-free) growth medium. Dynamic evolution of the headspace CO_2 and H_2 concentrations in (a) a batch initially charged with 60% headspace CO_2, (b) 40% CO_2, (c) 20% CO_2, and (d) 10% CO_2. Data presented clearly show that hydrogen can be produced only with CO_2 in acetate-free environments. Data are presented as mean values ± SD, error bars indicate standard deviations (n = 3, biologically independent samples). Source data are provided as a Source Data file.

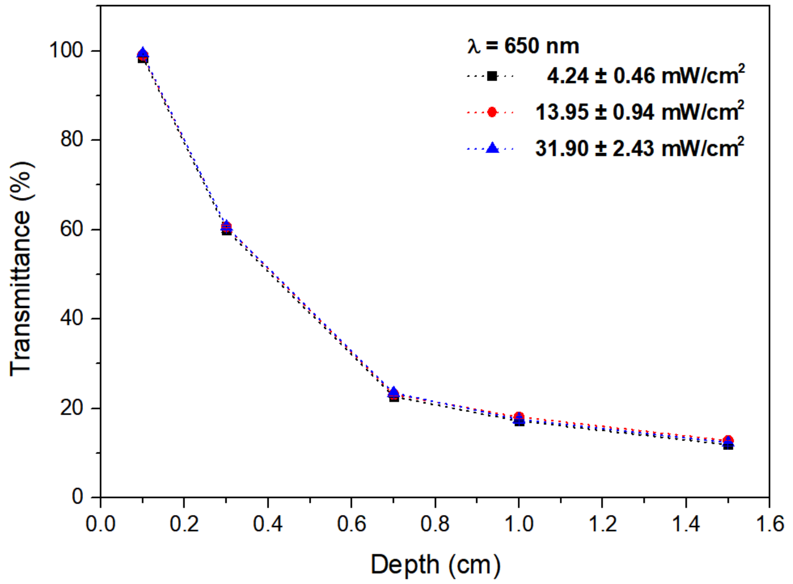

Supplementary Figure 23. Light transmittance within photobioreactor. Observe the light transmittance of the photobioreactor at light intensities of 4.24 mW/cm^2, 13.95 mW/cm^2 and 31.9 mW/cm^2 at a wavelength of 650 nm. Data are presented as mean values ± SD, error bars indicate standard deviations (n = 3, biologically independent samples). Source data are provided as a Source Data file.

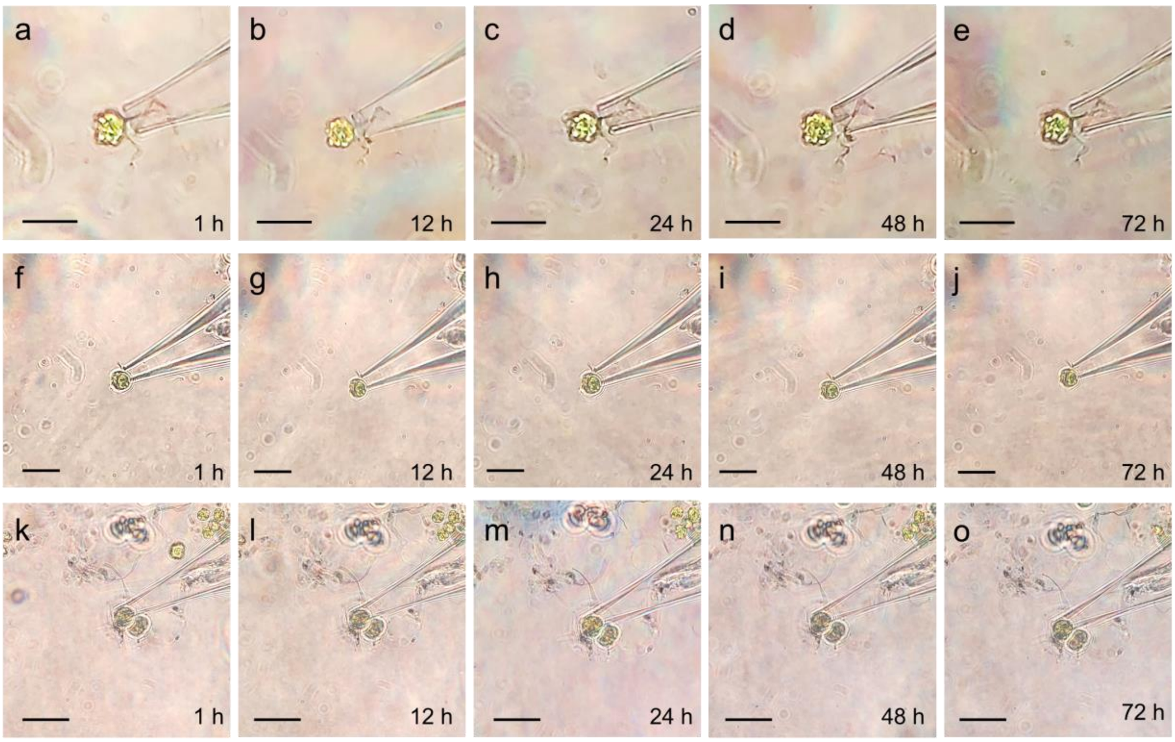

Supplementary Figure 24. Time-lapsed monitoring of *C. reinhardtii* - CNF. Tracking the cell division and fate of inserted CNF by optical microscopy with (a-e), (f-j) and (k-o). All relevant experiments were performed independently in triplicate with similar results.

Supplementary Table 1. Comparison of longevity and power density by biophotovoltaic literature reported system.

Microorganism	Type	Duration	Power density	Reference
Synechocystis sp. PCC6803	Biophotovoltaics	189 days	4.2 μW/cm^2	1
Synechococcus elongatus UTEX 2973	Biophotovoltaics	40 days	13.5 μW/cm^2	2
Synechocystis sp. PCC6803	Biophotovoltaics	4 days	0.001 μW/cm^2	3
Chlorella sp. UMACC 313	Biophotovoltaics	12 days	0.016 μW/cm^2	4
Synechococcus sp. WH 5701	Biophotovoltaics	32 days	0.002 μW/cm^2	5
Synechocystis sp. PCC6803	Micro biological solar cell	20 days	12 μW/cm^2	6
Synechocystis sp. PCC6803	Micro biological solar cell	3 days	0.06 μW/cm^2	7
Arthrospira maxima	Photosynthetic microbial fuel cell	8 days	0.001 μW/cm^2	8
Chlamydomonas reinhardtii	Photosynthetic electron extraction	50 days	12.1 μW/cm^2	This work

Supplementary Table 2. Power production of engineered cell culture as a function of batch total volumes. Considering the current extracted from single cells and the number of cells according to the culture volume. The unit is W.

	Cell culture volume (L)								
**Volume Ratio (%)**	**0.1**	**0.5**	**1**	**2**	**5**	**10**	**50**	**100**	**1000**
**10**	0.18	0.9	1.8	3.6	9.1	18	90	181	1814
**20**	0.36	1.8	3.6	7.2	18	36	181	363	3629
**30**	0.54	2.7	5.4	11	27	54	272	544	5443
**40**	0.72	3.6	7.2	15	36	72	363	726	7257
**50**	0.90	4.5	9.0	18	45	90	454	907	9072
**60**	1.2	5.4	11	21	54	108	544	1089	10887
**70**	1.4	6.3	13	25	63	127	635	1270	12700
**80**	1.6	7.2	15	29	72	145	726	1451	14515
**90**	1.8	8.1	18	33	81	163	816	1633	16329

Supplementary Table 3. Comparison of solar-to-fuel conversion performances by literature reported systems.

Photocatalyst	Microorganism	Type	Products	Duration	STF	Reference
G/TiO_2-001/101	-	PEC CO_2 reduction	CO, CH_4	4 hours	0.14%	9
TiO_2/N,Zn-Fe_2O_3/Cr_2O_3	-	PEC CO_2 reduction	HCOOH, CO, H_2	13 hours	0.15%	10
Pt-TiO_2/GaN/n^+-p Si	-	PEC CO_2 reduction	CO, H_2	10 hours	0.87%	11
Cs_2AgBiX_6	-	PEC CO_2 reduction	CO, CH_4	6 hours	0.04%	12
CdS NPs	*Escherichia coli*	Semiconductor material-microorganism hybrid	H_2	6 hours	0.11%	13
CdS NPs	*Moorella thermoacetica*	Semiconductor material-microorganism hybrid	CH_3COOH	4 days	2.44%	14
InP	*Saccharomyces cerevisiae Δzwf1*	Semiconductor material-microorganism hybrid	Shikimic acid	3 days	1.58%	15
Co-P|CoP_i	*Ralstonia eutropha*	Electrode-microbial hybrid	biomass and fusel alcohols	6 days	9.70%	16
TiO_2	Mixed culture (more than 15 species)	Electrode-microbial hybrid	CH_4	4 days	0.10%	17
Photosystem	*Chlamydomonas reinhardtii CC124*	Cell engineering	H_2	50 days	0.45%	This work

Supplementary Table 4. Comparison of the solar-to-hydrogen efficiencies and associated costs in relevant artificial photosynthesis systems reported in the literature.

Photocatalyst / Microorganism	Type	Duration (Total)	Solar-to-Hydrogen Efficiency	Total Hydrogen Evolved [a]	Cost (per 1g H_2 production)	Reference
InGaP/GaAs/GaInNAsSb triple-junction solar cell	Photovoltaic-electrolyzer (PV-E)	20 min (48 hours)	30%	46 mL	$28,041 [b]	18
Rh/TiO_2/oxide/AlInP-GaInP/GaInAs/GaAs tandem solar cell	Photovoltaic-electrochemical (PV-EC)	2.5 hours (20 hours)	19%	6.4 mL	$11,270 [c]	19
FTO/W:$BiVO_4$/Co-Pi-a-Si:H/nc-Si:H solar cell	Photovoltaic-PEC (PV-PEC)	1 hour	5.2%	0.03 mL	$817,063 [d]	20
Pt/$CuIn_{1-x}Ga_xSe_2$/CdS-nano-worm $BiVO_4$ cell	Dual photoelectrode (Dual-PE)	2 hours	3.7%	1.2 mL	$6,350 [e]	21
$SrTiO_3$:La-Rh/Au/$BiVO_4$:Mo sheet	Photocatalytic (PC)	13 hours	1.1%	3.1 mL	$1,147,041 [f]	22
Chlorella pyrenoidosa	Photobiological	7 days	-	0.09 mL	$172,231 [g]	23
Chlorella pyrenoidosa	Photobiological	3 days	-	0.05 mL	*$411,306* [h]	24
Chlorella pyrenoidosa	Photobiological	11 days	-	0.03 mL	*$162,670* [i]	25
Synechocystis PCC 6803	Photobiological	6 days	-	47 mL	*$12,086* [j]	26
Platymonas subcordiformis	Photobiological	12 hours	-	4.9 mL	*$2,361* [k]	27
Chlamydomonas reinhardtii tla1 CC4169	Photobiological	10 days	-	2.3 mL	$3,699 [l]	28
Chlamydomonas reinhardtii pgr5	Photobiological	14 days	-	2.8 mL	$87,095 [m]	29
Chlamydomonas reinhardtii	Photobiological	7 days	-	4 mL	$2,600 [n]	30
Chlamydomonas reinhardtii	Photobiological	26 days	-	23.3 mL	$9.586 [o]	31
Chlamydomonas reinhardtii	Artificial Photosynthesis	50 days	0.45%	90 mL	$1,194 [p]	This work

Note: Material cost estimation

a: The amounts of hydrogen fuel reported in each work by explicit quantification were listed.

b: Nafion 115 membrane 12.25 cm^2 ($0.34/$cm^2$, Fuel Cells Etc; US), Pt black 3.125 mg ($230/g, Premetek; US), Ir black 12.5 mg ($400/g, Premetek; US) Nafion resin solution (D-520) 5.64 µL ($3.34/mL, Ion Power; US), Carbon paper (GDL 28 BC) 5 cm^2 ($0.114/$cm^2$, Ion Power),

Ti mesh 5 cm^2 (\$9.43/cm^2, Sigma Aldrich; US), InGaP/GaAs/GaInNAsSb (unable to calculate the price). Photocurrent was driven for 48 h, whereas H_2 production was verified for 20 min. For extended fuel accrual, pressure management system should be required, leading to additional expense.
c: Rh 2.6 μg (\$762/g, Sigma Aldrich; US), TiO_2 4.54 μg (\$0.402/g, Sigma Aldrich; US), $Al_{0.35}In_{0.65}P$ 11.2 μg (\$21.42/g, averaged price, Sigma Aldrich; US), $Ga_{0.41}In_{0.59}P$ 869.1 μg (\$23.68/g, averaged price, Sigma Aldrich; US) $Ga_{0.89}In_{0.11}As$ 1.086 mg (\$18.79/g, averaged price, Sigma Aldrich; US) GaAs 74.4 mg (\$85.97/g, Sigma Aldrich; US) RuO_2 (unable to calculate the price)
d: W 0.221 μg (0.5% in average) (\$11.5/g, Sigma Aldrich; US) $BiVO_4$ 43.998 μg (99.5% in average) (\$0.902/g, Sigma Aldrich; US) FTO 25 cm^2 (\$0.0873/cm^2, Sigma Aldrich; US) nc-SiO_x 7.47 μg (\$1.782/g, SiO_2 nanopowder, Sigma Aldrich; US) nc-Si 118.7 μg (\$60/g, Si nanopowder, Sigma Aldrich; US) nc-SiC 2.73 μg (\$16/g, SiC nanopowder, Sigma Aldrich; US) a-Si 19.8 μg (\$0.405/g, Si powder, Sigma Aldrich; US) Pt counter, Co-Pi catalyst, Ag/Cr/Al back contact (unable to calculate the price)
e: Mo 9.87 mg (\$14.9/g, powder, Sigma Aldrich; US) $CuIn_{0.5}Ga_{0.5}Se_2$ 53.12 mg (\$18.455/g, averaged price, Sigma Aldrich; US) CdS 0.541 mg (\$9.96/g, Alfa Aesar; US) $NiSO_4$ 0.124 mg (\$7.57/g, Sigma Aldrich; US) $FeSO_4$ 1.215 mg (\$0.2/g, Sigma Aldrich; US) Pt, $BiVO_4$ (unable to calculate the price)
f: $SrCO_3$ 31.78 mg (\$5.01/g, Sigma Aldrich; US) TiO_2 17.19 mg (\$3.704/g, Sigma Aldrich; US) La_2O_3 1.46 mg (\$1.134/g, Sigma Aldrich; US) Rh_2O_3 1.14 mg (\$565/g, Sigma Aldrich; US) $Bi(NO_3)_3{\cdot}5H_2O$ 62.39 mg (\$0.256/g, Sigma Aldrich; US) V_2O_5 11.7 mg (\$0.246/g, Sigma Aldrich; US) MoO_3 9.3 μg (\$0.924/g, Sigma Aldrich; US) Au 185.1 mg (\$1,150/g, Sigma Aldrich; US) α-terpineol 260.4 mg (\$398.4/g, Sigma Aldrich; US) 2-(2-butoxyethoxy)ethanol 52.1 mg (\$0.0263/g, Sigma Aldrich; US) $RuCl_3{\cdot}3H_2O$ 44.45 μg (\$85/g, Sigma Aldrich; US) Acrylic resin SPB-TE1 104.2 mg (unable to calculate the price)
g: TAP 7 mL (\$50/L, UTEX; US), Dopamine 21 mg (\$1.288/g, Aladdin; China), Laccase 7 mg (\$139/g, Sigma Aldrich; US), Tannic acid 70 mg (\$0.485/g, Sigma Aldrich; US)
h: TAP 2 mL (\$50/L, UTEX; US), Dextran 19.2 mg (\$2.8/g, Sigma Aldrich; US), PEG solution 1.2 mL (\$1.34/mL, Sigma Aldrich; US), BSA solution 120 μL (\$0.62/mL, Sigma Aldrich; US)
i: TAP 8.5 mL (\$50/L, UTEX; US), Cationic Starch 75 mg (\$0.143/g, Sigma Aldrich; US)
j: BG11 400 mL (\$0.1268/mL, Sigma Aldrich; US)
k: CCCP 2.05 mg (\$504/g, Sigma Aldrich; US), Micronutrients (unable to calculate the price)
l: TAP 10 mL (\$50/L, UTEX; US), 5 mg Arginine (\$0.4/g, Sigma Aldrich; US), Sodium alginate 0.04 g (\$0.338/g, Sigma Aldrich; US), $CaCl_2$ 27.7 mg (\$0.672/g, Sigma Aldrich; US), Ar 65 mL (\$6.214/L, Sigma Aldrich; US)
m: TAP 10 mL (\$50/L, UTEX; US), Sodium ascorbate (AA) 198.11 mg (\$98.1/g, Sigma Aldrich; US), $CuSO_4$ 52 μg (\$0.35/g, Sigma Aldrich; US)
n: TAP 10mL (\$50/L, UTEX; US), Sodium alginate 0.04 g (\$0.338/g, Sigma Aldrich; US), $CaCl_2$ 27.7 mg (\$0.672/g, Sigma Aldrich; US), Ar 65 mL (\$6.214/L, Sigma Aldrich; US)
o: TAP 3 mL (\$50/L, UTEX; US), glucose 27.024 mg (\$0.0251/g, Sigma Aldrich; US), $Mg(OH)_2$ 8.748 mg (\$0.348/g, Sigma Aldrich; US), GO_x 0.3 KU (\$2.46/KU, Sigma Aldrich; US), CAT 3 mg (\$372/g, Sigma Aldrich; US)
p: TAP 150 mL (\$50/L, UTEX; US), Carbon nanofiber 3.3 mg (\$5/g, Sigma Aldrich; US), CTAB 0.67 mg (\$0.48/g, Sigma Aldrich; US), K_2PtCl_4 24.9 mg (\$83.6/g, Sigma Aldrich; US)

Supplementary Note 1. Calculation of penetration efficiency.

The LIVE/DEAD staining images were analyzed using ImageJ software, the penetration efficiency (P%) of the functionalized substrata can be quantified by the following equation:

$$\text{Penetration efficiency (\%)} = \frac{F_r \,/\, F_{rs}}{F_b \,/\, F_{bs}} \times 100\ \% \qquad \textbf{(1)}$$

where F_r and F_b represent the total cell fluorescence intensity in red (PI) and blue (DAPI) channels, respectively. F_rs and F_bs represent the fluorescence intensity per single cell in red and blue channels, respectively. The number (N_c) of cell captured on substrata can be calculated using the following equation:

$$N_\mathrm{C} = F_\mathrm{b}/F_\mathrm{bs} \qquad \textbf{(2)}$$

Supplementary Note 2. Calculation of photon to electron conversion efficiency per single cell.

Photosynthetic current extraction in single cellular photovoltaic power stations using SECM was calculated based on the equation below. The photon to electron conversion efficiency was determined by comparison of the current increase according to the light intensity:

$$\text{Photon to electron conversion efficiency per single cell (\%)} = \frac{n \times I_c \div F \div A_c}{\Phi_{ph}} \times 100\ \% \qquad \textbf{(3)}$$

where n is the number of electrons required for the redox mediator to conduct an electrochemical reaction. The redox mediator used in this paper is ferrocenemethanol, a one-electron reaction. I_c is the photosynthetic current increase according to the light intensity, F is the Faraday constant (96485 C/mol), A_c is the area of the cell surface that receives photons. Photons reached the cell perpendicularly, assuming the area A as an orthographic projection of the cell. The average cell area was estimated to be 78.54 μm^2. Φ_{ph} is the measured photon flux (μmols/m^2/s).

Supplementary Note 3. Calculation of cellular photovoltaic power.

Based on the electrons extracted from the photosynthetic electron transport chain, the power according to the batch volume was calculated based on the equation below. The difference between the maximum potential of the photosynthetic electron transport chain and the CNF surface potential is 1.3 V, and based on the 7.32 pA output current per cell, the following relationship holds:

$$\text{Photovoltaic power for batch volume (W)} = \frac{1.3\,V \times 7.32\,pA \times V_{Batch} \times P}{V_{Cell}} \qquad \textbf{(4)}$$

Where V_{Batch} is the total volume of engineered cell batch. P is the space factor occupied by cells in the total volume of the engineered cell batch. V_{Cell} is the volume of engineered cell that is spherical shape with a radius of 5 μm. At typical cell densities for *Chlamydomonas*, a power rating per volume can be deduced and presented in Table S1. Approximately a milk-

carton-sized batch (2000 mL) can power a typical laptop at 20-50 W.

Supplementary Note 4. Calculation of quantum yields for O_2 consumption and H_2 production.

Catalytic photosynthetic oxygen consumption and hydrogen production quantum yields were calculated based on the equation below. The reduction of one O_2 molecule to H_2O_2 and H_2O requires 3.5 electrons on average and the production of one H_2 molecule requires 2 electrons, giving the follow quantum yield equations:

$$\text{Quantum yield for gastight batch (\%, for } O_2 \text{ consumption)} = \frac{3.5 \times (N_{O_2}) \div t \div A_b}{\Phi_{ph}} \times 100\ \% \quad \textbf{(5)}$$

$$\text{Quantum yield for gastight batch (\%, for } H_2 \text{ production)} = \frac{2 \times (N_{H_2}) \div t \div A_b}{\Phi_{ph}} \times 100\ \% \quad \textbf{(6)}$$

where N_{O2} is the moles of consumed O_2 and N_{H2} is the moles of produced H_2. t is the reaction time to consume O_2 and to produce H_2, A_b is the area of the *C. reinhardtii* - CNF/Pt culture batch that receives photons. Photons reached the batch perpendicularly, assuming the area A_b as an orthographic projection of the batch. The average batch area was estimated to be 10 cm^2. Φ_{ph} is the measured photon flux of 108 μmols/m^2/s.

Supplementary Note 5. Oxygen reduction in competition with hydrogen evolution in the potential space.

Proton reduction is a pH dependent redox process that has a formal redox potential, $E^{0\prime}$, of 0 - (pH × 59) mV vs. the standard hydrogen electrode (SHE) (25 °C). The cell growth environment is optimal at pH 7, and therefore, the $E^{0\prime}$ of hydrogen evolution stays constant. Reduction of dissolved oxygen has a formal redox potential, $E^{0\prime}$, of - 330 mV vs. the standard hydrogen electrode (SHE) (25 °C), which varies as a function of the ratio between [O_2] and [$O_2^{\cdot-}$] as shown in equation (**9**) below.

$$2H^+ + 2e^- \rightarrow H_2 \qquad {}^7E^{0\prime} = -\ 0.41\ \text{V} \qquad \textbf{(7)}$$

$$O_2 + e^- \rightarrow O_2{\cdot-} \qquad {}^7E^{0\prime} = -\ 0.33\ \text{V} \qquad \textbf{(8)}$$

$$^7E \;=\; {}^7E^{0\prime} - \frac{0.059}{n} \log\frac{[O_2{\cdot-}]}{[O_2]} \qquad \textbf{(9)}$$

The oxygen concentration dropped below 5% after day 4, effectively placing oxygen reduction in competition with hydrogen evolution in the potential space. The amount of dissolved oxygen is 258 μM under the conditions of the cell culture medium at a temperature of 25 °C, 760 mmHg, and salinity of 0.1. An oxygen reduction reaction occurs steadily through the electrons extracted from the engineered algae, and the amount of dissolved oxygen decreases to 13 μM after the 8 days. The dissolved oxygen reduction potential is a Nernstian function of the ratio between O_2

and $O_2^{\cdot-}$ concentrations (**9**), decreased dissolved oxygen concentration pushed the formal potential cathodic up to -0.41 V.[52] Now the potential range is competitive to that of hydrogen evolution, and thus the competition autoregulated the oxygen content in the headspace.

Supplementary Note 6. Calculation of number of electrons exiting the alga-CNF/Pt power plants per day.

The maximum photosynthetic electron extraction to CNF from a single cell measured by SECM was 7.32 pA at 108 μmols/m[2]/s photons. Considering the volume and number of cells in the cell batch leads to the follow power production equation:

$$\text{Number of electrons exiting to CNF (mols)} = \frac{I_{ex} \times t \times N_{Cell}}{F} \qquad \textbf{(10)}$$

Where I_{ex} is the current extraction amount per single cell. t is the irradiation time per day. N_{Cell} is the total number of cells in cell culture. F is the faraday constant.

Supplementary Note 7. Proportion of H_2 produced by Pt and hydrogenase.

Many microalgae exhibit the ability to express hydrogenases under anaerobic conditions, enabling the reduction of protons into gaseous hydrogen. However, this phenomenon is constrained to stringent anaerobic growth environments. Algal [FeFe]-hydrogenases (HydA) play a central role in initiating hydrogen production through the oxidation of reduced ferredoxin (Fd), an electron mediator that becomes reduced by photosystem I and relies on the availability of reduced Fd. The two primary reactions follow distinct electron transport pathways: (i) HydA catalyzed hydrogen production and (ii) FNR (Ferredoxin-NADP$^+$ reductase) catalyzed NADPH production. According to findings from an in-vitro study, the introduction of FNR and NADP$^+$ to isolated thylakoids resulted in a substantial suppression over 75% of hydrogen production catalyzed by HydA.[32,33] This suppression is attributed to the inefficient interaction between HydA and Fd in the presence of FNR. Importantly, the rate of Fd electron movement is approximately FNR 7: HydA 1. In essence, the interaction between FNR and PSI introduces kinetic limitations that hinder effective electron transfer to HydA, thereby impacting hydrogen production.

This study highlights that only a minute fraction of electrons, bypassing the wire-based pathway, follow the photosynthetic route to reach Fd. The majority approximately 85% preferentially engage in the process of carbon fixation through FNR. Consequently, the likelihood of these stray electrons eventually reaching HydA is exceedingly challenging, leading to a significantly negligible ratio. Given the distribution of electron flow, it is expected that hydrogen production via inorganic catalysts would substantially outweigh the contribution from HydA, aligning with the data presented in Figure 4b. Furthermore, measurements of single-cell current extraction rates through SECM demonstrated extraction rates of up to 90% under a light intensity of 108 μmol/m^2/s (Figure 2e). These findings bolster the concept of hydrogen production through electron extraction along the wire. Considering a 90% electron

extraction efficiency in the lead wire and a 15% electron path efficiency from Fd to HydA, the electron utilization ratio for HydA is estimated to be around 1.5% at most. As a strategic approach to optimizing hydrogenase utilization within this context, the fusion of Fd and HydA emerges as a viable consideration.

Supplementary Note 8. Co-culture of engineered cells and pristine cells for sustainable photosynthetic hydrogen production.

One of the unique properties of aquatic algae is that during normal, healthy growth, some of the organic matter fixed by photosynthesis is released into the surrounding water.[34] Much research has been done on the release of dissolved organic matter (DOM) from phytoplankton and its composition.[35-39] DOM is released into the external environment across the plasma membrane with a concentration gradient several orders of magnitude of the organic matter. Figures for algal DOM emissions in nature are widely reported, and dissolved organic carbon (DOC) values can range from 0 to 80% of those fixed by photosynthesis. Most report typical values in the range of 5–30%.[40,41,42] Algal exudates are composed of a wide range of compounds, but are usually carbohydrate-dominant. They range from simple monosaccharides to larger heteropolysaccharides, and their chemical composition can vary considerably.[43,44,45] Soluble algae products (SAPs) can be absorbed into the surrounding culture community, and are therefore known to influence cell metabolism.[46,47,48] SAPs play important roles in the microalgae community as electron sources or metabolism promoters. Recently, a cross-feeding study has been conducted to increase photosynthetic activity and increase biomass production through SAPs leaked out through heterogeneous co-cultivation.[49,50] In our study, total organic carbon measurement confirmed an increase in SAPs, mainly composed of carbohydrates, and co-culture of engineered cells and pristine cells allowed continuous production of hydrogen even after acetate was consumed.

Supplementary references

1. Bombelli, P. *et al.* Powering a microprocessor by photosynthesis. *Energy Environ. Sci.* **15**, 2529–2536 (2022).
2. Zhu, H. et al. Development of a longevous two-species biophotovoltaics with constrained electron flow. *Nat. Commun.* **10**, 4282 (2019).
3. Sawa, M. et al. Electricity generation from digitally printed cyanobacteria. *Nat. Comm.* **8**, 1327–1337 (2017).
4. Ng, F. L., Phang, S. M., Periasamy, V., Yunus, K. & Fisher, A. C. Enhancement of power output by using alginate immobilized algae in biophotovoltaic devices. *Sci. Rep.* **7**, 1–8 (2017).
5. McCormick, A. J. et al. Photosynthetic biofilms in pure culture harness solar energy in a mediatorless bio-photovoltaic cell (BPV) system. *Energy Environ. Sci.* **4**, 4699–4709 (2011).
6. Liu, L. & Choi, S. Self-sustainable, high-power-density bio-solar cells for lab-on-a-chip applications. *Lab. Chip* **17**, 3817–3825 (2017).
7. Lee, H. & Choi, S. A micro-sized bio-solar cell for self-sustaining power generation. *Lab Chip* **15**, 391–398 (2015).
8. Inglesby, A. E., Yunus, K. & Fisher, A. C. *In situ* fluorescence and electrochemical monitoring of a photosynthetic microbial fuel cell. *Phys. Chem. Chem. Phys.* **15**, 6903–6911 (2013).
9. Xiong, Z. *et al.* Synthesis, characterization and enhanced photocatalytic CO_2 reduction activity of graphene supported TiO_2 nanocrystals with coexposed {001} and {101} facets. *Phys. Chem. Chem. Phys.* **18**, 13186–13195 (2016).
10. Sekizawa, K., Sato, S., Arai, T. & Morikawa, T. Solar-driven photocatalytic CO_2 reduction in water utilizing a ruthenium complex catalyst on p-type Fe_2O_3 with a multiheterojunction. *ACS Catal.* **8**, 1405–1416 (2018).
11. Chu, S. *et al.* Photoelectrochemical CO_2 reduction into syngas with the metal/oxide interface. *J. Am. Chem. Soc.* **140**, 7869–7877 (2018).
12. Liu, Z. *et al.* Synthesis of lead-free Cs_2AgBix_6 (X = Cl, Br, I) double perovskite nanoplatelets and their application in CO_2 photocatalytic reduction. *Nano Lett.* **21**, 1620–1627 (2021).
13. Honda, Y., Shinohara, Y., Watanabe, M., Ishihara, T. & Fujii, H. Photo-biohydrogen production by photosensitization with biologically precipitated cadmium sulfide in hydrogen-forming recombinant *Escherichia coli*. *ChemBioChem* **21**, 3389–3397 (2020).
14. Sakimoto, K. K., Wong, A. B. & Yang, P. Self-photosensitization of nonphotosynthetic bacteria for solar-to-chemical production. *Science* **351**, 74–77 (2016).
15. Guo, J. *et al.* Light-driven fine chemical production in yeast biohybrids. *Science* **362**, 813–816 (2018).
16. Liu, C., Colón, B. C., Ziesack, M., Silver, P. A. & Nocera, D. G. Water splitting–biosynthetic system with CO_2 reduction efficiencies exceeding photosynthesis. *Science* **352**, 1210–1214 (2016).
17. Fu, Q. *et al.* Hybrid solar-to-methane conversion system with a Faradaic efficiency of up to 96%. *Nano Energy* **53**, 232–239 (2018).
18. Jia, J. et al. Solar water splitting by photovoltaic-electrolysis with a solar-to-hydrogen

efficiency over 30%. *Nat. Commun.* **7**, 13237 (2016).
19. Cheng, W.-H. et al. Monolithic photoelectrochemical device for direct water splitting with 19% efficiency. *ACS Energy Lett.* **3**, 1795–1800 (2018).
20. Han, L. et al. Efficient water-splitting device based on a bismuth vanadate photoanode and thin-film silicon solar cells. *ChemSusChem* **7**, 2832–2838 (2014).
21. Kobayashi, H. et al. Development of highly efficient $CuIn_{0.5}Ga_{0.5}Se_2$-based photocathode and application to overall solar driven water splitting. *Energy Environ. Sci.* **11**, 3003–3009 (2018).
22. Wang, Q. et al. Scalable water splitting on particulate photocatalyst sheets with a solar-to-hydrogen energy conversion efficiency exceeding 1%. *Nat. Mater.* **15**, 611–615 (2016).
23. Su, D. *et al.* Enzyme-modulated anaerobic encapsulation of chlorella cells allows switching from O_2 to H_2 production. *Angew. Chem.* **131**, 4032–4035 (2019).
24. Xu, Z. *et al.* Photosynthetic hydrogen production by droplet-based microbial micro-reactors under aerobic conditions. *Nat. Commun.* **11**, 1–10 (2020).
25. Chen, J. *et al.* Chemical flocculation-based green algae materials for photobiological hydrogen production. *ACS Appl. Bio Mater.* **5**, 897–903 (2022).
26. Touloupakis, E., Margarita, A., Benavides, S., Cicchi, B. & Torzillo, G. Growth and hydrogen production of outdoor cultures of *Synechocystis* PCC 6803. *Algal Res.* **18**, 78–85 (2016).
27. Ran, C., Yu, X., Jin, M. & Zhang, W. Role of carbonyl cyanide m-chlorophenylhydrazone in enhancing photobiological hydrogen production by marine green alga *Platymonas subcordiformis*. *Biotechnol. Prog.* **22**, 438–443 (2006).
28. Kosourov, S. N., Ghirardi, M. L. & Seibert, M. A truncated antenna mutant of *Chlamydomonas reinhardtii* can produce more hydrogen than the parental strain. *Int. J. Hydrogen Energy* **36**, 2044–2048 (2010).
29. Khosravitabar, F. & Hippler, M. A new approach for improving microalgal biohydrogen photoproduction based on safe & fast oxygen consumption. *Int. J. Hydrogen Energy* **44**, 17835–17844 (2019).
30. Kosourov, S. N. & Seibert, M. Hydrogen photoproduction by nutrient-deprived *Chlamydomonas reinhardtii* cells immobilized within thin alginate films under aerobic and anaerobic conditions. *Biotechnol. Bioeng.* **102**, 50–58 (2009).
31. Chen, J. *et al.* Engineering a chemoenzymatic cascade for sustainable photobiological hydrogen production with green algae. *Energy Environ. Sci.* **13**, 2064–2068 (2020).
32. Yacoby, I. et al. Photosynthetic electron partitioning between [FeFe]-hydrogenase and ferredoxin: NADP+-oxidoreductase (FNR) enzymes in vitro. *Proc. Natl Acad. Sci. USA* **108**, 9396–9401 (2011).
33. Nagarajan, D., Dong, C. Di, Chen, C. Y., Lee, D. J. & Chang, J. S. Biohydrogen production from microalgae—Major bottlenecks and future research perspectives. *Biotechnol. J.* **16**, 1–12 (2021).
34. Puddu, A. *et al.* Bacterial uptake of DOM released from P-limited phytoplankton. *FEMS Microbiol. Ecol.* **46**, 257–268 (2003).
35. Wetz, M. S. & Wheeler, P. A. Release of dissolved organic matter by coastal diatoms. *Limnol. Oceanogr.* **52**, 798–807 (2007).
36. Flynn, K. & Butler, I. Nitrogen sources for the growth of marine microalgae: role of dissolved free amino acids. *Mar. Ecol. Prog. Ser.* **34**, 281–304 (1986).

37. Loh, A. N. & Bauer, J. E. Distribution, partitioning and fluxes of dissolved and particulate organic C, N and P in the eastern North Pacific and Southern Oceans. *Deep. Res. Part I Oceanogr. Res. Pap.* **47**, 2287–2316 (2000).
38. Søndergaard, M. *et al.* Net accumulation and flux of dissolved organic carbon and dissolved organic nitrogen in marine plankton communities. *Limnol. Oceanogr.* **45**, 1097–1111 (2000).
39. Flynn, K. J., Clark, D. R. & Xue, Y. Modeling the release of dissolved organic matter by phytoplankton. *J. Phycol.* **44**, 1171–1187 (2008).
40. Malinsky-Rushansky, N. Z. & Legrand, C. Excretion of dissolved organic carbon by phytoplankton of different sizes and subsequent bacterial uptake. *Mar. Ecol. Prog. Ser.* **132**, 249–255 (1996).
41. Hansell, D. A. & Carlson. C. A. *Biogeochemistry of Dissolved Organic Matter* 2nd edn, Ch. 2 (Academic Press, Oxford, 2015).
42. Biersmith, A. & Benner, R. Carbohydrates in phytoplankton and freshly produced dissolved organic matter. *Mar. Chem.* **63**, 131–144 (1998).
43. Hulatt, C. J. & Thomas, D. N. Dissolved organic matter (DOM) in microalgal photobioreactors: A potential loss in solar energy conversion? *Bioresour. Technol.* **101**, 8690–8697 (2010).
44. Yu, Y. *et al.* Accumulation characteristics of soluble algal products (SAP) by a freshwater microalga *Scenedesmus* sp. LX1 during batch cultivation for biofuel production. *Bioresour. Technol.* **110**, 184–189 (2012).
45. Zhuang, L. L. *et al.* Soluble Algal Products (SAPs) in large scale cultivation of microalgae for biomass/bioenergy production: A review. *Renew. Sustain. Energy Rev.* **59**, 141–148 (2016).
46. Leflaive, J. & Ten-Hage, L. Chemical interactions in diatoms: Role of polyunsaturated aldehydes and precursors. *New Phytol.* **184**, 794–805 (2009).
47. Zhang, T. Y., Yu, Y., Wu, Y. H. & Hu, H. Y. Inhibitory effects of soluble algae products (SAP) released by *Scenedesmus* sp. LX1 on its growth and lipid production. *Bioresour. Technol.* **146**, 643–648 (2013).
48. Mandalam, R. K. & Palsson, B. O. *Chlorella vulgaris* (Chlorellaceae) does not secrete autoinhibitors at high cell densities. *Am. J. Bot.* **82**, 955–963 (1995).
49. Wu, Y. H. *et al.* Mixed cultivation as an effective approach to enhance microalgal biomass and triacylglycerol production in domestic secondary effluent. *Chem. Eng. J.* **328**, 665–672 (2017).
50. Kong, L. *et al.* Cross-feeding among microalgae facilitates nitrogen recovery at low C/N. *Environ. Res.* **211**, 113052 (2022).
51. Bard, A. J. & Mirkin, M. V. *Scanning Electrochemical Microscopy* (CRC Press, 2012).
52. Bard, A. J. & Faulkner, L. R. *Electrochemical Methods: Fundamentals and Applications* (Wiley, 2001).
