# Supplementary material for: Prolonged hydrogen production by engineered green algae photovoltaic power stations
Source: Nat Commun. 2023 Oct 25;14:6768. doi: 10.1038/s41467-023-42529-3 (PMC10600337; doi:10.1038/s41467-023-42529-3)
Supplement: Supplementary file 3 — Description of Additional Supplementary Files [file 41467_2023_42529_MOESM3_ESM.pdf]

Description of Additional Supplementary Files

Supplementary Movie 1

Video footage of a fuel cell powered RC vehicle driven directly by injection of photosynthetic hydrogen. A comparison clip to the RC car powered by pure hydrogen is also included.
