# Supplementary material for: Prolonged hydrogen production by engineered green algae photovoltaic power stations
Source: Nat Commun. 2023 Oct 25;14:6768. doi: 10.1038/s41467-023-42529-3 (PMC10600337; doi:10.1038/s41467-023-42529-3)
Supplement: Supplementary file 5 — Reporting Summary [file 41467_2023_42529_MOESM5_ESM.pdf]

nature portfolio

Corresponding author(s): WonHyoung Ryu, Hyun S. Ahn

Last updated by author(s): Oct 6, 2023

Reporting Summary

Nature Portfolio wishes to improve the reproducibility of the work that we publish. This form provides structure for consistency and transparency in reporting. For further information on Nature Portfolio policies, see our Editorial Policies and the Editorial Policy Checklist.

Statistics

For all statistical analyses, confirm that the following items are present in the figure legend, table legend, main text, or Methods section.

n/a	Confirmed	
☐	☒	The exact sample size (*n*) for each experimental group/condition, given as a discrete number and unit of measurement
☐	☒	A statement on whether measurements were taken from distinct samples or whether the same sample was measured repeatedly
☒	☐	The statistical test(s) used AND whether they are one- or two-sided<br>*Only common tests should be described solely by name; describe more complex techniques in the Methods section.*
☐	☒	A description of all covariates tested
☐	☒	A description of any assumptions or corrections, such as tests of normality and adjustment for multiple comparisons
☐	☒	A full description of the statistical parameters including central tendency (e.g. means) or other basic estimates (e.g. regression coefficient) AND variation (e.g. standard deviation) or associated estimates of uncertainty (e.g. confidence intervals)
☒	☐	For null hypothesis testing, the test statistic (e.g. *F*, *t*, *r*) with confidence intervals, effect sizes, degrees of freedom and *P* value noted<br>*Give P values as exact values whenever suitable.*
☒	☐	For Bayesian analysis, information on the choice of priors and Markov chain Monte Carlo settings
☐	☒	For hierarchical and complex designs, identification of the appropriate level for tests and full reporting of outcomes
☒	☐	Estimates of effect sizes (e.g. Cohen's *d*, Pearson's *r*), indicating how they were calculated

Our web collection on statistics for biologists contains articles on many of the points above.

Software and code

Policy information about availability of computer code

Data collection	30 W halogen lamp (SZ2-CLS, Olympus, Japan) was used for illumination, and the intensity of the light was measured by an optical power meter (8230E-82311B, ADC Corp., Japan). The number of cells per mL was determined by measuring the absorbance at 750 nm (OD750) and by performing a trypan blue assay for cell counting. Optical microscopy was performed on an NSM-3B microscope (SAMWON, South Korea). Scanning electron microscopy (SEM) was conducted on a JSM-7610F-Plus (JEOL, Japan) equipped with an energy-dispersive spectrometer. Confocal laser scanning microscopy measurement was carried out using a Carl Zeiss LSM 980 laser confocal microscope. The pH values were determined by using a pH meter (Thermo Orion Star A211 pH Benchtop, Thermo Scientific™) equipped with a micro sensor (Mettler Toledo, Thermo Scientific™). For organic material analysis, nuclear magnetic resonance (NMR) AVANCE II 400 (Bruker Biospin) was used. The absorption spectrum was measured on a JASCO V-770 UV-vis spectrophotometer. Cell viability was assessed using CellTiterGlo® (Promega, G7572). All electrochemical analyses were conducted on a CHI920D SECM bipotentiostat (CH Instruments, USA). Gas quantification was determined Agilent 7890B gas chromatograph equipped with a thermal conductivity detector (TCD) and a Carboxen 1000 12 ft column (Supelco).
Data analysis	The counting of cell numbers was carried out using Image J 1.53e. Data process was conducted using Origin 9.

For manuscripts utilizing custom algorithms or software that are central to the research but not yet described in published literature, software must be made available to editors and reviewers. We strongly encourage code deposition in a community repository (e.g. GitHub). See the Nature Portfolio guidelines for submitting code & software for further information.

Data

Policy information about availability of data

All manuscripts must include a data availability statement. This statement should provide the following information, where applicable:

- Accession codes, unique identifiers, or web links for publicly available datasets
- A description of any restrictions on data availability
- For clinical datasets or third party data, please ensure that the statement adheres to our policy

The authors declare that all relevant data supporting the finding of this study are available within this paper and its Supplementary Information files. If specific data is believed to be missing, that data is available from the corresponding authors upon request.

Research involving human participants, their data, or biological material

Policy information about studies with human participants or human data. See also policy information about sex, gender (identity/presentation), and sexual orientation and race, ethnicity and racism.

Reporting on sex and gender	Not applicable in this study.
Reporting on race, ethnicity, or other socially relevant groupings	Not applicable in this study.
Population characteristics	Not applicable in this study.
Recruitment	Not applicable in this study.
Ethics oversight	Not applicable in this study.

Note that full information on the approval of the study protocol must also be provided in the manuscript.

Field-specific reporting

Please select the one below that is the best fit for your research. If you are not sure, read the appropriate sections before making your selection.

☒ Life sciences ☐ Behavioural & social sciences ☐ Ecological, evolutionary & environmental sciences

For a reference copy of the document with all sections, see nature.com/documents/nr-reporting-summary-flat.pdf

Life sciences study design

All studies must disclose on these points even when the disclosure is negative.

Sample size	No sample size calculation was performed and they are described in the figure legend. All experiments were performed with at least three replicates; the results obtained suggested the chosen sample sizes were appropriate because clear distinctions could be seen with the current sample size.
Data exclusions	No data were excluded from the analyses.
Replication	Each experiment was performed at more than three independent replications and was described in the figure legend.
Randomization	Algal cells were randomly swimming with operating photosynthetic power plant. Enough replications and control of algal cell engineering were carried out in consistent environment to control covariates.
Blinding	Blinding is not possible in this study of green algal cells.

Reporting for specific materials, systems and methods

We require information from authors about some types of materials, experimental systems and methods used in many studies. Here, indicate whether each material, system or method listed is relevant to your study. If you are not sure if a list item applies to your research, read the appropriate section before selecting a response.

Materials & experimental systems

n/a	Involved in the study
☒	☐ Antibodies
☐	☒ Eukaryotic cell lines
☒	☐ Palaeontology and archaeology
☒	☐ Animals and other organisms
☒	☐ Clinical data
☒	☐ Dual use research of concern
☒	☐ Plants

Methods

n/a	Involved in the study
☒	☐ ChIP-seq
☒	☐ Flow cytometry
☒	☐ MRI-based neuroimaging

Eukaryotic cell lines

Policy information about cell lines and Sex and Gender in Research

Cell line source(s)	Chlamydomonas reinhardtii strain CC-4348 (sta 6-1 mt+, from the Chlamydomonas Resource Center) was used in all experiments.
Authentication	The cell line was authenticated by the Chlamydomonas Resource Center.
Mycoplasma contamination	The cell line was not tested for Mycoplasma contaminations.
Commonly misidentified lines (See ICLAC register)	NA
